# Lipids and Phosphorylation Conjointly Modulate Complex Formation of β_2_-Adrenergic Receptor and β-arrestin2

**DOI:** 10.3389/fcell.2021.807913

**Published:** 2021-12-23

**Authors:** Kristyna Pluhackova, Florian M. Wilhelm, Daniel J. Müller

**Affiliations:** Department of Biosystems Science and Engineering, Eidgenössische Technische Hochschule (ETH) Zurich, Basel, Switzerland

**Keywords:** GPCR, arrestin, phosphorylation, biased signalling, molecular dynamics simulations, acidic lipids, Martini2 parametrization, intracellular loop 3

## Abstract

G protein-coupled receptors (GPCRs) are the largest class of human membrane proteins that bind extracellular ligands at their orthosteric binding pocket to transmit signals to the cell interior. Ligand binding evokes conformational changes in GPCRs that trigger the binding of intracellular interaction partners (G proteins, G protein kinases, and arrestins), which initiate diverse cellular responses. It has become increasingly evident that the preference of a GPCR for a certain intracellular interaction partner is modulated by a diverse range of factors, e.g., ligands or lipids embedding the transmembrane receptor. Here, by means of molecular dynamics simulations of the β_2_-adrenergic receptor and β-arrestin2, we study how membrane lipids and receptor phosphorylation regulate GPCR-arrestin complex conformation and dynamics. We find that phosphorylation drives the receptor’s intracellular loop 3 (ICL3) away from a native negatively charged membrane surface to interact with arrestin. If the receptor is embedded in a neutral membrane, the phosphorylated ICL3 attaches to the membrane surface, which widely opens the receptor core. This opening, which is similar to the opening in the G protein-bound state, weakens the binding of arrestin. The loss of binding specificity is manifested by shallower arrestin insertion into the receptor core and higher dynamics of the receptor-arrestin complex. Our results show that receptor phosphorylation and the local membrane composition cooperatively fine-tune GPCR-mediated signal transduction. Moreover, the results suggest that deeper understanding of complex GPCR regulation mechanisms is necessary to discover novel pathways of pharmacological intervention.

## Introduction

Cells react to extracellular stimuli with the help of a complex protein machinery in the plasma membrane. A major component of this sensory machinery is the class of seven-transmembrane-spanning proteins known as G protein-coupled receptors (GPCRs) (Rosenbaum et al., 2009). GPCRs bind extracellular ligands and relay the signal across the cell membrane through interactions with G proteins and arrestins on the intracellular side (Lefkowitz, 2004). Heterotrimeric G proteins are activated by binding to the intracellular binding pocket of the active receptor, which facilitates exchange of GDP for GTP on the α subunit of the G protein and subsequent dissociation of the α and βγ subunits, which afterwards trigger a multitude of signaling pathways (McCudden et al., 2005). After phosphorylation of the C-terminus and in some cases also of the intracellular loops of the active GPCR by G protein-coupled receptor kinases (GRKs) (Pitcher et al., 1998; Gurevich and Gurevich, 2019), arrestin can bind the receptor. Arrestin either desensitizes the receptor and promotes its recycling or degradation (Benovic et al., 1987) and modulates diverse signaling pathways (Lefkowitz and Shenoy, 2005). Recent studies (Marshall, 2016; Thomsen et al., 2016; Nguyen et al., 2019; Nguyen and Lefkowitz, 2021) have discovered the formation of so-called megaplexes of GPCR, G protein and arrestin, which supports sustained signaling from internalized receptors.

GPCRs interact with signaling molecules through distinct structural elements. The highly dynamic, disordered and little conserved intracellular loop 3 (ICL3) and C-terminus of the receptors have been reported to play important roles in binding G proteins, GRKs and arrestins (Chee et al., 2008; Komolov et al., 2017; Yin et al., 2019; Seyedabadi et al., 2021). The dynamics of these disordered regions was shown to be reduced by the presence of interacting proteins, suggesting a disorder-to-order transition. Nevertheless, the structure of the disordered regions could not be resolved so far with the exception of receptors having a short ICL3 or C-terminus, like rhodopsin (5DGY) (Zhou et al., 2016), formyl peptide receptor 2 (6LW5) (Chen et al., 2020) or cryoTEM structures of the metabotropic glutamate receptor (7MTS) (Seven et al., 2021) or CC chemokine receptor 5 (7O7F) (Isaikina et al., 2021), both complexed with Gi protein. In fact, rhodopsin is up to date the only GPCR with a completely resolved structure (Okada et al., 2004). However, improvements in computational efficiency over the last decades have made molecular dynamics (MD) simulations capable of capturing the unstructured loop regions (Srivastava et al., 2020) and helped unravelling the importance of ICL3 for the active state of the β_2_-adrenergic receptor (β_2_AR), a prominent GPCR responsive to adrenaline (Ozcan et al., 2013; Bruzzese et al., 2018). Such structural plasticity and adaptability of the ICL3 and C-terminus could explain the promiscuous binding of 20 different α subunits of G proteins, 7 GRKs and only two arrestins to the cytosolic binding pocket of more than 800 different GPCRs.

Due to their vital role in signal transduction, GPCRs are key regulators in healthy and disease states and are therefore targeted by one third of all current drugs (Hauser et al., 2018). Most of these drugs act as unbiased ligands and therefore only shift the probability of the extent of receptor activation and do not specifically activate one or the other signaling pathway (Weis and Kobilka, 2014). However, GPCR pharmacology is more complex and more diverse mechanisms modulating GPCR signaling have been discovered over the last years. Modulators of receptor signaling stabilize distinct receptor conformations, which preferentially signal through one or the other G protein or through arrestins. This modulatory mechanism is called biased signaling (Seyedabadi et al., 2019; Gurevich and Gurevich, 2020).

One typical modulator of GPCR signaling are the lipids comprising biological membranes (Manna et al., 2019; Sejdiu and Tieleman, 2020). Thereby, biased signaling can result from different (co)localization of GPCRs, G proteins, GRKs and arrestin (Seyedabadi et al., 2019), from changes in the oligomerization state of the receptor (Gahbauer and Böckmann, 2016; Periole, 2017) and from differences in the GPCR conformational flexibility (Zocher et al., 2012; Manna et al., 2016). The most important lipidic modulators discovered so far are cholesterol (Paila and Chattopadhyay, 2010; Pluhackova et al., 2016; Gahbauer et al., 2018; Kiriakidi et al., 2019), polyunsaturated fatty acids (PUFAs) (Guixà-González et al., 2016) and acidic lipids (Dijkman and Watts, 2015). The latter were also found to be essential for the complex formation of the β_2_AR with the kinase GRK5 (Komolov et al., 2017) and thus for the phosphorylation of the receptor (Komolov et al., 2017). Moreover, acidic lipids can influence signaling pathways by determining which G protein preferentially binds the respective GPCR (Strohman et al., 2019). Negatively charged lipids also influence the activation state of the receptor (Dawaliby et al., 2016) and recent MD simulations have suggested two possible underlying mechanisms. Firstly, negatively charged lipids can intercalate between transmembrane helices (TM) 6 and 7 and block the ionic lock interaction that stabilizes the inactive state of the receptor (Neale et al., 2015; Bruzzese et al., 2020). Alternatively, attachment of ICL3 to a negatively charged membrane surface was suggested to contribute to the stabilization of the outward tilt of TM6 in the active state of the receptor (Bruzzese et al., 2018; Díaz et al., 2019). Moreover, acidic lipids were proven to be important for the binding of arrestin to rhodopsin (Sommer et al., 2006).

Other means of signal modulation are protein post-translational modifications (PTM), of which phosphorylation is the most common among GPCRs. The localization and the extent of phosphorylation depend on the kinase, the experimental conditions (*in-vitro* or *in-vivo*), and the agonist type (Trester-Zedlitz et al., 2005). Different phosphorylation patterns evoke different physiological and pathophysiological responses (Butcher et al., 2012; Yang et al., 2015). Recently, large scale MD simulations (Latorraca et al., 2020) and comparison of experimentally resolved structures of arrestin in complex with different GPCRs or phosphorylated peptides (Kim and Chung, 2020) have revealed that different phosphorylation patterns lead to distinct arrestin conformations. This finding suggests that arrestin adjusts its shape for optimal interaction with different downstream signaling partners such as clathrin, MAPK, and others.

Here, we study by extensive atomistic MD simulations how phosphorylation and native acidic lipids concomitantly modulate the structure and dynamics of the β_2_AR. We further investigate the role of receptor phosphorylation and membrane composition in arrestin binding to the β_2_AR. The results suggest that the interplay between phosphorylation and local membrane composition fine-tune GPCR-mediated signaling.

## Results and Discussion

### Receptor Phosphorylation and Membrane Composition Steer ICL3 to the Membrane Surface

**FIGURE 1:**
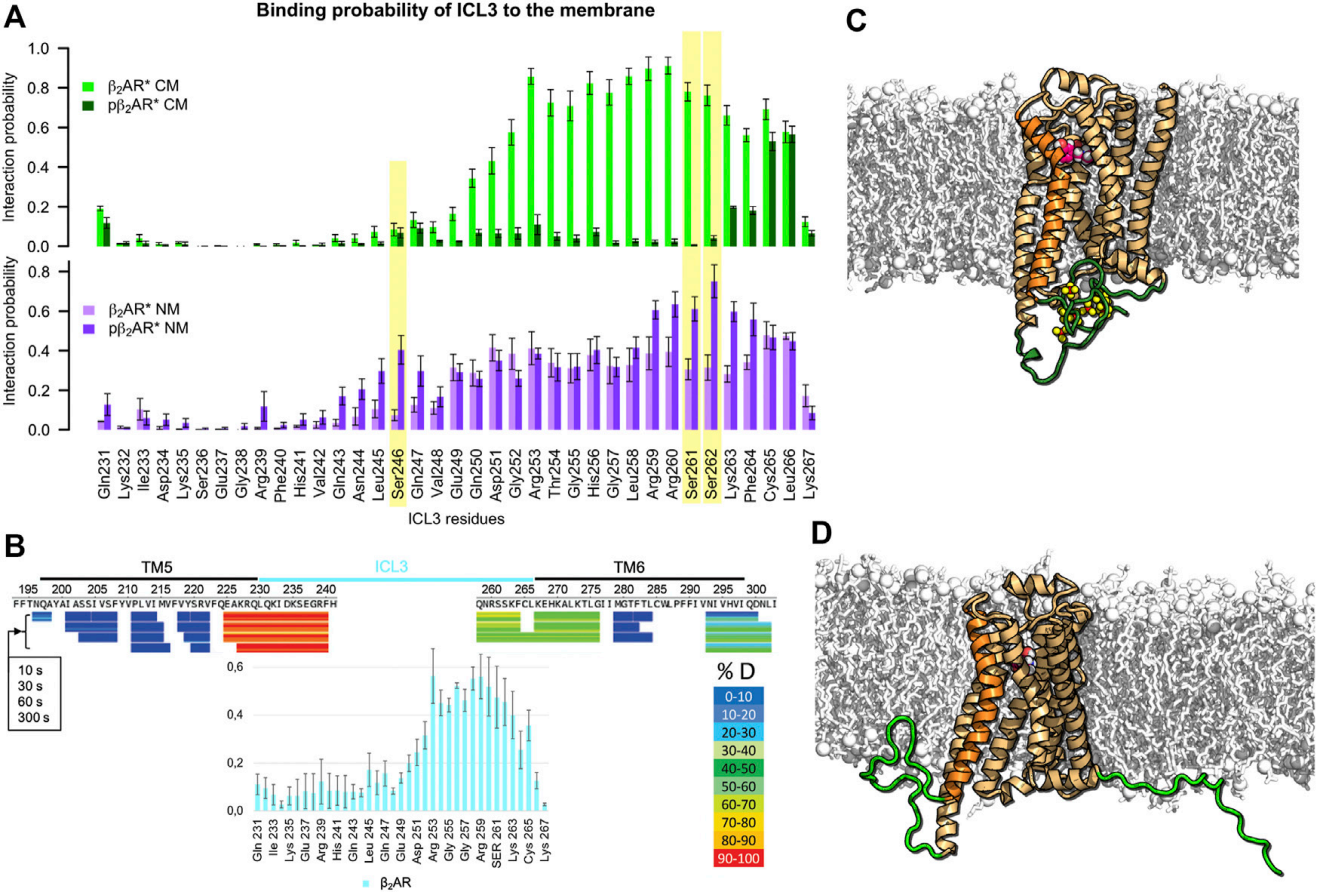
Interactions of ICL3 with membrane depend on β_2_AR phosphorylation and lipid composition. **(A)** Probability of ICL3 interactions with charged (CM, green colors) and neutral (NM, purple colors) lipid membranes for active β_2_AR* in different phosphorylation states (probabilities for the nonphosphorylated receptor in light and phosphorylated in dark colors). Yellow background indicates residues that are phosphorylated in the respective data sets. Interaction probabilities were acquired from three independent REST MD simulations per lipid membrane composition and β_2_AR* phosphorylation state. Error bars give standard error of the mean over 6 analyzed time intervals (100–150 ns and 150–200 ns of each REST MD simulation at 310 K). **(B)** Comparison of the ICL3-membrane interaction probability in the inactive, nonphosphorylated state of β_2_AR (cyan bar plot at the bottom) embedded in the charged membrane to the hydrogen-deuterium exchange experiments on β_2_AR inactivated by carazolol (adapted with permission from (Zhang et al., 2010), copyright 2010 American Chemical Society). The lower hydrogen-deuterium exchange (shown in green) at the cytoplasmic end of TM6 and the proximal ICL3 hint to reduced water accessibility as compared to the cytoplasmic end of TM5 and the adjacent part of ICL3 (shown in red/orange colors). Hydrogen-deuterium exchange times of 10, 30, 60 and 300 s are shown. **(C)** Phosphorylated active pβ_2_AR* and **(D)** Nonphosphorylated active β_2_AR*. The receptor, displayed as orange cartoon with TM6 highlighted in brighter orange, is embedded in a negatively charged membrane (white sticks and spheres) consisting of DOPC, DOPG and cholesterol. ICL3 and C-terminus are highlighted in green, phosphorylated residues are shown as yellow/red spheres. Adrenaline is shown as magenta/white spheres.

First, we quantified the binding of the intracellular loops and the C-terminus of the β_2_AR to the membrane in dependence of receptor phosphorylation and lipid membrane composition by estimating the minimal distance between each residue and the membrane over the simulation. Interaction probability reflects the portion of the analyzed simulation time in which the residue is found closer than 0.5 nm to the lipids (a distance reflecting direct interactions and interactions mediated by a single water molecule). To ensure sufficient sampling of the highly dynamic ICL3 and C-terminus, replica exchange solute tempering (REST) simulations were performed. The comparison of membrane binding probabilities obtained in free and REST MD simulations is shown in Supplementary Figure S1. Figure 1A, top shows that the nonphosphorylated ICL3 of the β_2_AR in complex with the agonist adrenaline (β_2_AR*) binds to the negatively charged membrane composed of negatively charged 1,2-dioleoyl-sn-glycero-3-phosphoglycerol (DOPG), zwitterionic 1,2-dioleoyl-sn-glycero-3-phosphocholine (DOPC), and cholesterol (36:54:10 molar ratio). This finding agrees with previous MD simulations of the β_2_AR, adenosine A1 receptor, and cannabinoid receptor type 1 in single-component lipid membranes (Bruzzese et al., 2018; Díaz et al., 2019; Bruzzese et al., 2020). Interestingly, only the TM6-proximal half of ICL3 is membrane attached. Hydrogen-deuterium exchange experiments of carazolol-bound β_2_AR, which showed significantly lower exchange of hydrogen at the TM6-proximal region of ICL3 compared to the TM5-proximal region (Zhang et al., 2010), support our observation (Figure 1B). Moreover, the probability of ICL3 to interact with the membrane is higher in the active (adrenaline-bound) state of the receptor than in the inactive (ICI 118551-bound) state (Supplementary Figure S2). A similar pattern, albeit with slightly higher interaction probabilities, is found also for β_2_AR* complexed with the G_s_ protein, embedded in the same membrane (Supplementary Figure S3).

**FIGURE 2:**
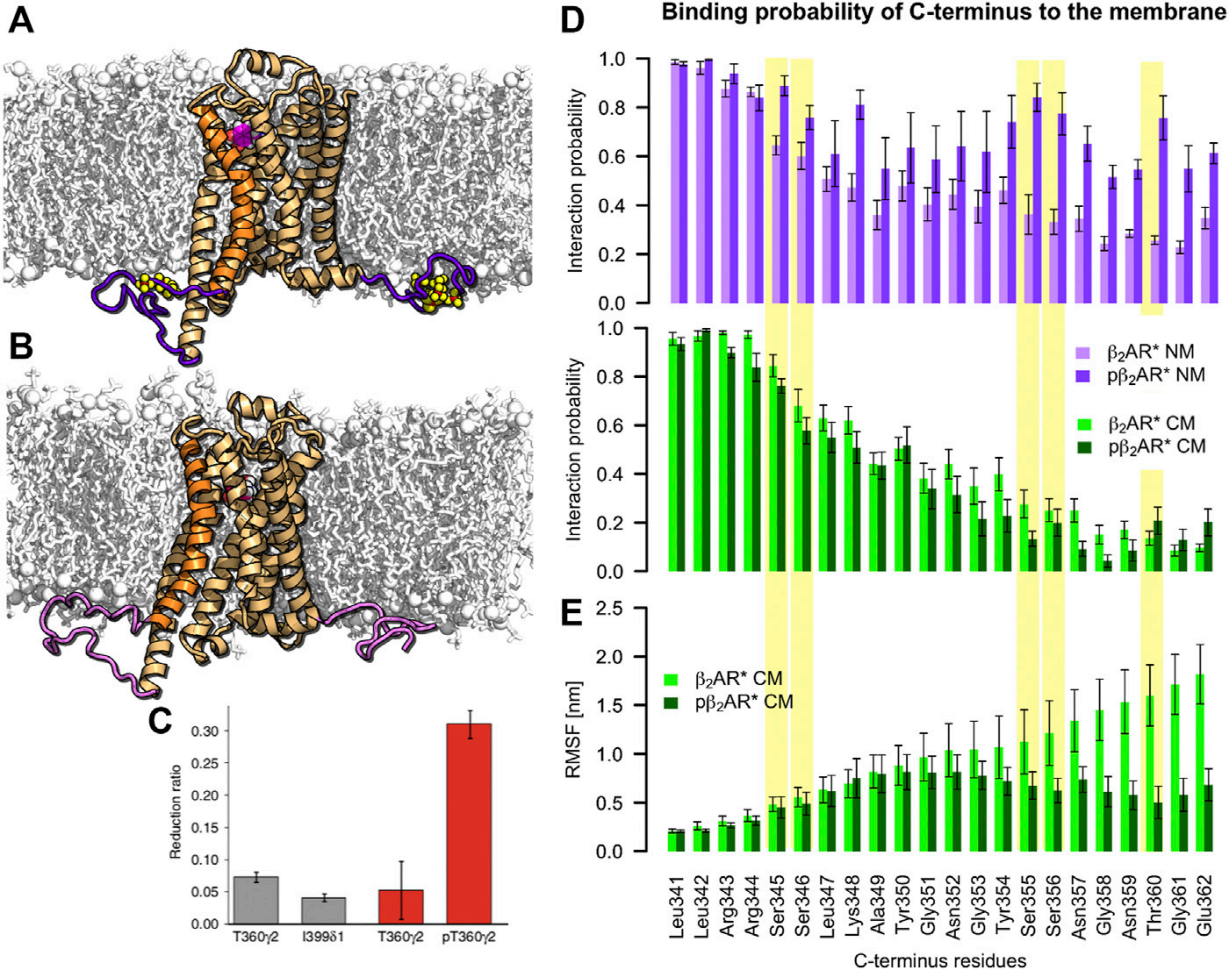
C-terminus interactions with the negatively charged membrane are independent of β_2_AR phosphorylation. **(A)** Phosphorylated active pβ_2_AR* and **(B)** nonphosphorylated active β_2_AR* (orange cartoon with TM6 highlighted in brighter orange, ICL3 and C-terminus are highlighted in (light) purple, phosphorylated residues are shown as yellow/red spheres) embedded in a neutral membrane (white sticks and spheres) consisting of DOPC, DOPE and cholesterol. Adrenaline is shown as magenta/white spheres. **(C)** NMR observation of Shiraishi et al. showing phosphorylation induced decrease of flexibility of the C-terminus of β_2_AR* with full agonist formoterol in POPC:POPG 3:2 lipid nanodiscs (Shiraishi et al., 2018). **(D)** Probability of C-terminus interactions with the neutral (NM, purple colors) and charged (CM, green colors) membranes for active β_2_AR* in different phosphorylation states (nonphosphorylated in light and phosphorylated in dark colors). Yellow background indicates residues that are phosphorylated in the respective data sets. **(E)** Root mean square fluctuations (RMSF) per residue along the C-terminus of active β_2_AR* pre and post phosphorylation determined over 500-ns intervals of standard MD simulations after exclusion of the first 500 ns for equilibration purposes. Error bars denote the SEM [N (β_2_AR*) = 5, N (pβ_2_AR*) = 9].

Upon phosphorylation of ICL3 at residues S246, S261 and S262 (Komolov et al., 2017), the interaction of the loop with acidic lipids is abolished and ICL3 is liberated into the cytoplasm (Figure 1A, top). Figures 1C,D visualize typical conformations of ICL3 and C-terminus in phosphorylated (pβ_2_AR*) and nonphosphorylated states of β_2_AR*, respectively. A similar reduction of membrane contacts is observed for the receptor in the inactive state (Supplementary Figure S2). A lack of acidic lipids reverses the effect of phosphorylation, i.e., in the nonphosphorylated state of β_2_AR*, the TM6-proximal half of ICL3 interacts with neutral lipids with a probability of ≈40% only. This probability increases to 60–80% for phosphorylated residues and their neighboring residues (Figure 1A, bottom). Exemplary snapshots of both pβ_2_AR* and β_2_AR* embedded in a neutral membrane are shown in Figures 2A,B.

### Phosphorylation Does Not Influence C-Terminus Availability in the Cytosol

The probability of the C-terminus binding to the membrane surface decreases with increasing distance from the membrane inserted receptor core. At a distance of 10 amino acids from helix 8 (H8), i.e., at G351, the C-terminus attaches to the acidic membrane surface in only ≈40% of the simulation time. At a distance of 20 amino acids from H8, i.e., at G361, the interaction probability drops to ≈10% (Figure 2D bottom). While in a negatively charged membrane phosphorylation of the C-terminus does not change this behavior, in a neutral membrane the phosphorylated C-terminus interacts more frequently with the uncharged membrane surface (Figure 2D top). Thus, the ability of the phosphorylated C-terminus to bind arrestin in the cytosol and attract it to the receptor core is likely to be reduced in the absence of native acidic lipids. We therefore propose that acidic lipids play an important role in complex formation of pβ_2_AR* with arrestin by repelling the phosphorylated C-terminus from the membrane surface and thereby preserving its availability in the cytosol for arrestin binding.

In nuclear magnetic resonance (NMR) measurements of β_2_AR* activated by the full agonist formoterol and reconstituted in 1-palmitoyl-2-oleoyl-sn-glycero-3-phosphocholine (POPC): 1-palmitoyl-2-oleoyl-sn-glycero-3-phosphoglycerol (POPG) (3:2) nanodiscs, Shiraishi and colleagues have shown that receptor phosphorylation reduces the flexibility of the helix 8-proximal C-terminus (i.e., residues L341-T360) (Shiraishi et al., 2018) (Figure 2C). It was proposed that the reduced flexibility results from the attachment of the C-terminus to the membrane. Here, comparison of root mean square fluctuations (RMSF) per residue of the active β_2_AR* before and after phosphorylation also shows smaller flexibility of the C-terminus in the phosphorylated state (Figure 2E). However, our simulations show that the binding probability of the C-terminus to the negatively charged membrane is independent of the phosphorylation (Figure 2D). Instead, the reduced flexibility of the phosphorylated C-terminus arises from interactions of the C-terminus with itself and the receptor body. Indeed, analysis of average minimal distance between C-terminal residues with the receptor (Supplementary Figure S4) shows that while the C-terminus residues in the nonphosphorylated state do not interact with other parts of β_2_AR, the phosphorylated C-terminus contacts helix 8 or self-interacts (phosphorylated serines in positions 344 and 345 cluster often *via* Na^+^ with phosphorylated serines in position 355 and 356). Therefore, our simulations suggest an alternative interpretation of the experimentally observed reduction in β_2_AR* C-terminus flexibility upon phosphorylation (Shiraishi et al., 2018).

### Phosphorylation and Membrane Lipids Influence the Extent of Receptor Activation

**FIGURE 3:**
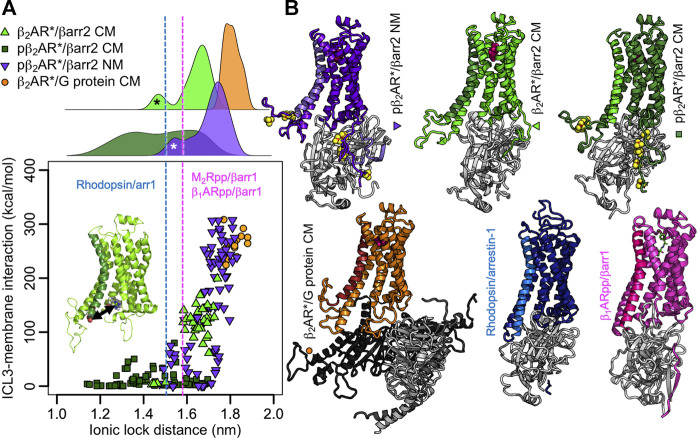
ICL3-membrane binding influences the extent of β_2_AR* activation. **(A)** Absolute interaction energies between the ICL3 of (p)β_2_AR* and the membrane plotted against the ionic lock distance (distance of Cα atoms of Arg131 and Glu268, highlighted in the inset). Each data point equals an average over a 100-ns-interval. The first 500 ns of each simulation were excluded for equilibration purposes. Data represents 5 β_2_AR*/βarr2 CM simulations, 7 pβ_2_AR*/βarr2 CM simulations, 7 pβ_2_AR*/βarr2 NM simulations, 3 β_2_AR*/G protein CM simulations. The ionic lock distances from the rhodopsin/arrestin-1 (Zhou et al., 2016), β_1_ARpp/βarr1 (Lee et al., 2020), and M_2_Rpp/βarr1 (Staus et al., 2020) structures are highlighted as blue and magenta dotted lines, respectively. On the top, the distributions of the ionic lock distances are shown. The peaks highlighted by asterisks point to simulations in which the ICL3 attached to arrestin instead to the membrane in one β_2_AR*/βarr2 CM simulation (black asterisk) and two pβ_2_AR*/βarr2 NM simulations (white asterisk). The phosphate groups were omitted in the interaction energy calculation to obtain comparable values for phosphorylated and nonphosphorylated receptors. **(B)** Representative structures of the complexes shown as cartoons. The receptor is color coded according to the simulation type, with TM6 highlighted in different color shades and the phosphates shown as yellow/red spheres. Arrestin is shown in light grey, G_s_ protein in different shades of grey. The membrane and solvent were omitted from the visualization for clarity. The structures of the complexes of rhodopsin with arrestin-1 (Zhou et al., 2016) and β_1_ARpp/βarr1 (Lee et al., 2020) are shown for comparison.

The NMR investigations of Shiraishi et al. further show that after phosphorylation, TM6 of the formoterol-activated β_2_AR* moves inwards (Shiraishi et al., 2018), suggesting a closer contact with arrestin than with the G protein. Indeed, in the crystal structure of adrenaline-bound β_2_AR* with a G_s_ protein complex (3SN6) (Rasmussen et al., 2011), visualized in Figure 3, the ionic lock distance (i.e., the distance between the Cα of R131 and Cα of E268) measures 1.73 nm. In the arrestin-bound structures, the ionic lock distance shortens and ranges from 1.47 nm for the rhodopsin/*all-trans*-retinal/arrestin-1 complex (5DGY) (Zhou et al., 2016), to 1.58 nm for the chimera of the M_2_ muscarinic receptor core and the C-terminus of vasopressin 2 receptor (M_2_Rpp)/iperoxo/βarr1 (6U1N) (Staus et al., 2020) and 1.59 nm for the chimera of the β_1_-adrenergic receptor core and the C-terminus of vasopressin 2 receptor β_1_ARpp/formoterol/βarr1 complex (6TKO) (Lee et al., 2020) (Figure 3). As the presence of both ligand and intracellular binding partners influence the extent of activation (Weis and Kobilka, 2014), we have modelled complexes of β-arrestin2 (βarr2) and β_2_AR* in different phosphorylated states and equilibrated them for microseconds in the same neutral or negatively charged membranes we used above (Supplementary Table S1; Figure 3). Plotting the ionic lock distance against the absolute value of the interaction energy of ICL3 with the membrane reveals that complexes with higher ICL3-membrane interaction strength exhibit longer ionic lock distances (Figure 3). As expected from the above results, the phosphorylated ICL3 in the pβ_2_AR*/βarr2 complex detaches from the negatively charged membrane. Thereby the ionic lock distribution varies widely hinting to different bound states of arrestin. In the nonphosphorylated state, the ICL3 in the β_2_AR*/βarr2 complex interacts half as strongly with the membrane as nonphosphorylated ICL3 in the β_2_AR*/G_s_ protein complex. Moreover, the ionic lock distance in the β_2_AR*/βarr2 complex varies only moderately with the most probable distances located between those observed in the crystal/cryoEM structures of complexes with arrestin and with the G_s_ protein. Neutralization of the membrane charge attracts the phosphorylated ICL3 in the pβ_2_AR*/βarr2 complex to the membrane surface and increases the ionic lock distance almost to the extent of the fully open active receptor complexed with the G_s_ protein. It is interesting to note that in one β_2_AR*/βarr2 complex in the charged membrane and in two complexes of pβ_2_AR*/βarr2 in the neutral membrane, the ICL3 attached to arrestin instead of the membrane, thus leading to smaller interaction strength with the membrane and shorter ionic lock distances (the corresponding peaks in the ionic lock distance histograms in Figure 3 are highlighted by asterisks). The broad distributions of the ionic lock distances in β_2_AR complexes with βarr2 indicate their large flexibility and dynamics, which is investigated in the next section.

### Receptor Phosphorylation and Membrane Lipids Determine Arrestin Binding Mode and Dynamics

The five structures of GPCRs with arrestin published so far (Huang et al., 2020; Lee et al., 2020; Staus et al., 2020; Yin et al., 2019; Zhou et al., 2016) show remarkable variability in insertion depth of arrestin into the receptor core, the structure of arrestin’s fingerloop, and the orientation of arrestin relative to the receptor. Both the M_2_Rpp (Staus et al., 2020) and the β_1_ARpp (Lee et al., 2020), each modified with the C-terminus of the vasopressin 2 receptor (V_2_R), in complex with β-arrestin1 (βarr1) in nanodiscs show similar orientation of arrestin compared to rhodopsin/arrestin-1 (Zhou et al., 2016), although the fingerloop is only helical in the rhodopsin/arrestin-1 complex. It is important to mention here that the native M_2_R lacks phosphorylatable residues on the C-terminus and instead is phosphorylated on ICL3. The inclusion of the C-terminus of the V_2_R could thus result in different orientation of arrestin in M_2_Rpp as compared to wild-type M_2_R, which holds true also for the β_1_ARpp construct. GPCRs are divided into two classes with respect to arrestin binding (Oakley et al., 2000). Complexes of class A receptors (all rhodopsin-like GPCRs including β_2_AR and β_1_AR) with preferentially β-arrestin2 are transient and class A receptors resensitize quickly. Class B receptors (e.g., V_2_R, angiotensin II type 1 receptor, the oxytocin receptor and the neurotensin 1 receptor) form stable complexes with either of the β-arrestins and resensitize slowly. Swapping of the C-termini was shown to convert class A receptors into class B and vice versa (Oakley et al., 1999). Thus the β_1_ARpp/βarr1 (Lee et al., 2020) was made stable enough for structure determination, however, it must not necessarily represent the native form of the complex. In our analysis arrestin inserts deepest into the β_1_ARpp and least deep in the M_2_Rpp. The two independently resolved structures of the neurotensin receptor 1 with β-arrestin1 in detergent (Yin et al., 2019; Huang et al., 2020) show significantly different orientations of arrestin relative to the receptor (rotated by ≈ 90° and strongly tilted). The authors admit that this strongly tilted orientation, resulting from interactions between the C-edge of arrestin and the detergent micelle, may be exaggerated due to the small size of the detergent micelle (Huang et al., 2020). We therefore compare our results mainly to the complexes of M_2_Rpp, β_1_ARpp, and rhodopsin with arrestins.

**FIGURE 4:**
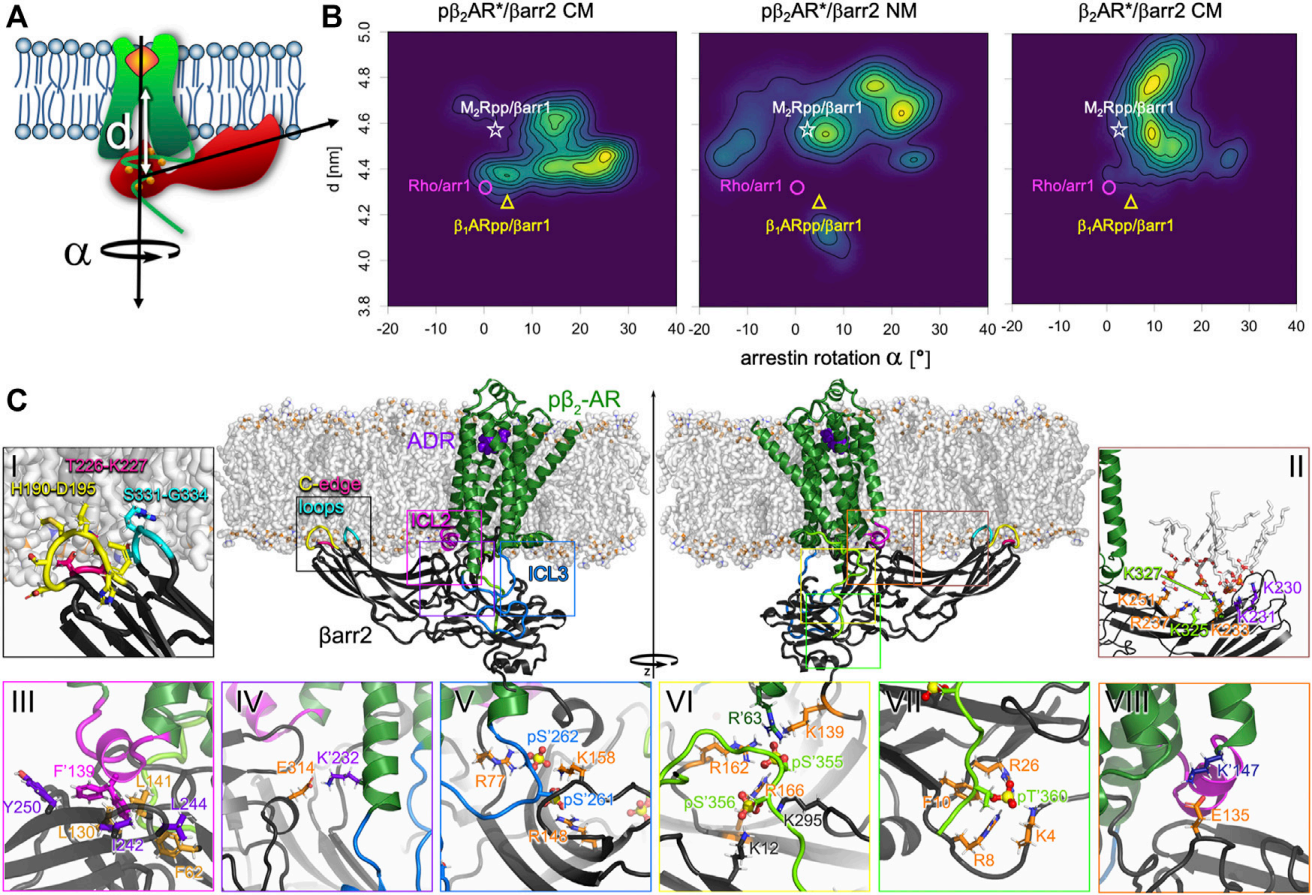
Phosphorylation and membrane composition influence binding orientation of βarr2 relative to (p) β_2_AR*. **(A)** Definition of binding characteristics used to describe arrestin binding distance d (vertical distance between the center of mass of the transmembrane helix bundle of the receptor and of the center of mass of the upper 4 beta sheets in arrestin’s N-lobe) and arrestin rotation α around the membrane normal relative to the arrestin orientation in the rhodopsin/arrestin-1 complex. **(B)** 2D densities of the binding distance d and arrestin rotation α of phosphorylated activated pβ_2_AR*/βarr2 complex in the charged (CM) and neutral membrane (NM) and of nonphosphorylated activated β_2_AR*/βarr2 complex in the CM. For comparison d and α were evaluated for experimentally resolved structures of M_2_Rpp/βarr1 (Staus et al., 2020) (white star), rhodopsin/arrestin-1 (Zhou et al., 2016) (magenta circle), and β_1_ARpp/βarr1 (Lee et al., 2020) (yellow triangle). **(C)** Key interactions of the pβ_2_AR*/βarr2 complex. Side views of the adrenaline-bound (purple spheres) pβ_2_AR* (green cartoon with ICL2 in magenta, ICL3 in blue and C-terminus in light green) embedded in a charged membrane (white sticks) in complex with βarr2 (black cartoon with membrane inserted C-edge loops in yellow, hotpink, and cyan). The insets show the interactions between βarr2 and pβ_2_AR* and βarr2 and the membrane which were most often observed in the simulations. Receptor residues are marked with an apostrophe (‘) to distinguish them from arrestin residues. Phosphates are shown as spheres with yellow phosphorus and red oxygen atoms. Residues of interest are shown as sticks with differently colored carbons (for sake of better visibility) and atom-type coded nitrogen (blue), oxygen (red), hydrogen (white), and sulphur (golden). Inset **(i)**: Arrestin’s C-edge loops H190-D195 (yellow), T226-K227 (hotpink) and S331-G334 (cyan) protrude deep into the membrane. Inset **(ii)**: DOPG lipids (shown as white sticks with orange phosphates and red oxygens) bind to the highly electropositive well of arrestin’s C-lobe. Residues experimentally determined to bind phosphatidylinositol lipids (Gaidarov et al., 1999) and hexakisphosphate (Milano et al., 2006) (K233, R237, K251) are colored orange, two additional residues (K325 and K327) known to bind hexakisphosphate (Milano et al., 2006) are colored green, and two further lysines (K230 and K231) detected here as additional interaction partners of DOPG are colored purple. Inset **(iii)**: F’139 (magenta) in the ICL2 of the pβ_2_AR* inserts into the crevice between the N- and C-lobe of arrestin, interacting mainly with Y250, I242, L244 (purple), and L130, L141 and F62 (orange). Two possible positions of F′139 are shown. Inset **(iv)**: Salt bridge formed between K′232 (purple) on TM5 of the receptor and E314 (orange) of the arrestin. Inset **(v)**: The phosphorylated residues pS′262 and pS′261 (blue sticks) of the receptor’s ICL3 (blue cartoon) often interact with the positively charged amino acids on arrestin, here R77, K158 and R148 (orange). Inset **(vi)**: Phosphorylated residues pS′355 and pS′356 on the C-terminus of the receptor (green backbone) are forming salt bridges with a number of positively charged amino acids. Here, pS′356 interacts with K12, K295 (black), and R166 (orange). Latter is also bound to pS′356, which further interacts with R162, K139 (orange) in arrestin and R′63 (dark green) on the receptor’s ICL1. Inset **(vii)**: The phosphorylated pT′360 (green) is involved in a number of salt bridges, here with K4, R8, and R26 (orange). Moreover, it exerts a large force on F10 (orange sticks). Inset **(viii)**: The salt bridge between K′147 (dark blue) from the intracellular end of TM3 and E135 (orange) in the middle loop of arrestin.

We determined β-arrestin2 insertion depth d (calculated as the distance along the membrane normal between the receptor core and the N-lobe of arrestin) and rotation α relative to the β_2_AR* in three different simulation types (for visualization of the parameters see Figure 4A). β-arrestin2 inserts the deepest into the phosphorylated active pβ_2_AR* in presence of acidic lipids (d = 4.48 ± 0.02 nm). The average distance to the receptor core in the nonphosphorylated receptor in a charged membrane and of the phosphorylated receptor in a neutral membrane was slightly increased, amounting to 4.65 ± 0.06 and 4.64 ± 0.04 nm, respectively. Moreover, in one simulation of β_2_AR*/βarr2 in the charged membrane and in two simulations of pβ_2_AR*/βarr2 in the neutral membrane β-arrestin2 unbound from the core of the receptor as indicated by a distance d > 5.3 nm (data not shown). Such a movement of arrestin away from the receptor core possibly hints to a preference for a tail-engaged arrestin-GPCR complex, mainly in case of the pβ_2_AR*/βarr2 in the neutral membrane as the phosphorylated C-terminus is likely to stay firmly bound to the arrestin.

The 2D density plots of d versus α in Figure 4B reveal the complex conformation and flexibility in more detail. In the case of the phosphorylated receptor embedded in the charged membrane (Figure 4B, left), three interconnected complex orientations, which differ by 30° rotation of arrestin and by approximately 0.4 nm difference in insertion depth, were found. This range of arrestin-receptor distance between 4.3 and 4.7 nm reflects well the insertion depths of arrestin in the crystal/cryoEM structures (i.e., rhodopsin/arrestin-1 4.33 nm (Zhou et al., 2016), β_1_ARpp/βarr1 4.25 nm (Lee et al., 2020) and M_2_Rpp/βarr1 4.57 nm (Staus et al., 2020)). While in one complex conformation the arrestin rotation of 0–10° matches perfectly the experimental rotations [rhodopsin/arrestin-1 1.5° (Zhou et al., 2016), β_1_ARpp/βarr1 6.4° (Lee et al., 2020), and M_2_Rpp/βarr1 3.3° (Staus et al., 2020)], the other two conformations gave larger rotation angles. If the receptor lacks phosphorylation (Figure 4B, right), arrestin orients in a single rotation mode amounting to approximately 10°, however, with two insertion depths. The orientation of the tighter complex resembles the looser complex type of the phosphorylated receptor and M_2_Rpp/βarr1 (Staus et al., 2020). In the other complex arrestin is shifted by 0.3 nm further away from the receptor. The lack of negatively charged lipids has a large impact on the complex of the phosphorylated receptor and arrestin (Figure 4B, middle). Firstly, the variance of the rotation angle increases significantly, i.e., arrestin reaches a rotational angle from −20° to +30°. Secondly, in most complexes arrestin is less deeply inserted. It is interesting to note that the small density at d∼4.1 nm and α∼8° represents a conformation in which the phosphorylated ICL3 attached firmly on arrestin’s N-lobe instead of binding to the neutral membrane surface.

Complex dynamics was quantified as average of the standard deviations of binding distance d and angle α over 500 ns analysis intervals. The steadiest complex is pβ_2_AR/βarr2 (variation of insertion depth of 0.065 ± 0.005 nm and angle α of 3.5 ± 0.2°). Neutralization of the membrane environment leads to slightly increased dynamics (variation of insertion depth of 0.071 ± 0.005 nm and angle α of 4.2 ± 0.4°). Lack of phosphorylation increases the dynamics of the complex more than membrane neutralization (variation of insertion depth of 0.104 ± 0.018 nm and angle α of 4.2 ± 0.3°).

Taken together with the observations from the previous section that the ionic lock distance is increased and ICL3 attached to the membrane surface in pβ_2_AR*/βarr2 in a neutral membrane and in β_2_AR*/βarr2 in a charged membrane, we conclude that binding of ICL3 to the membrane surface leads to less tight binding of arrestin and the receptor.

### The Complexes of (p)β_2_AR* with βarr2 are Stabilized by Manifold Interactions

In order to identify the elements that stabilize the complexes of (p)β_2_AR* with β-arrestin2, we have investigated the interactions between the receptor, arrestin and the membrane in more detail. Our simulations have shown that in all complexes the tip of the C-edge of arrestin inserts into the membrane (Figure 4Ci), which is in agreement with cryoEM structures (Lee et al., 2020; Sommer et al., 2006), fluorescence quenching experiments and previous MD simulations (Lally et al., 2017). Supplementary Figure S5 shows that H190-D195, T226-K227, and S331-G334 are membrane attached for more than 90% of the time in all simulations. Moreover, negatively charged DOPG lipids attached to the positively charged amino acids, K230, K231, K233, R237, K251, K325 and K327 at the well of the C-lobe of arrestin (Figure 4Cii; Supplementary Figure S5). On average 3.5 ± 0.3 and 3.3 ± 0.5 DOPG lipids are bound at those seven lysines or arginines in pβ_2_AR*/βarr2 and β_2_AR*/βarr2, respectively. Interestingly, three out of those positively charged residues, i.e., K233, R237 and K251, were discovered to bind phosphatidylinositol lipids by mutagenetic experiments (Gaidarov et al., 1999). Moreover, residues from β-arrestin1 corresponding to K233, R237, K251, K324 and K326 of β-arrestin2 were shown by X-ray crystalography to bind inositol hexakisphosphate (Milano et al., 2006) and were suggested to interact with phosphatidylinositol lipid headgroups by a docking study (Milano et al., 2002). Taken together with the increased arrestin rotational flexibility in the neutral membrane, we conclude that negatively charged DOPG lipids restrict arrestin’s dynamics by attaching the highly electropositive well of arrestin’s C-lobe to the membrane surface. By doing so, acidic DOPG lipids substitute the function of the highly negatively charged phosphatidylinositol lipids.

Our simulations have revealed that the ICL2 of the receptor is helical (in β_2_AR*/βarr2 CM are 5.0 ± 0.6 ICL2 residues helical, in pβ_2_AR*/βarr2 CM 6.1 ± 0.2, and in pβ_2_AR*/βarr2 NM 4.9 ± 0.2; averages over 500 ns intervals ± SEM). Such ICL2 helicity resembles that in the crystal and cryoEM structures [rhodopsin/arrestin-1 6 residues (Zhou et al., 2016), β_1_ARpp/βarr1 6 residues (Lee et al., 2020), and M_2_Rpp/βarr1 7 residues (Staus et al., 2020)]. The ICL2 sits in the crevice between the N- and C-lobe of arrestin with F139 exerting repulsive forces on arrestin residues L141, I242, as well as on F62, L244, L130, and Y250 (Figure 4Ciii). This observation suggests that the function of F139 is to restrict the insertion depth of arrestin into the receptor.

Moreover, residue-residue forces (Supplementary Figures S7–S11) and interaction probabilities of individual residue pairs (Supplementary Datasheet S2) between arrestin and the receptor revealed that apart from interactions of arrestin’s fingerloop with the receptor and of the phosphorylated ICL3 and C-terminus with arrestin (Figures 4Cv–vii), two additional ion pairs stabilize the complex. In detail, E314 of arrestin interacts with K232 in the receptor’s TM5 (Figure 4Civ) and E135 of arrestin is bound to K147 in TM3 (Figure 4Cviii).

**FIGURE 5:**
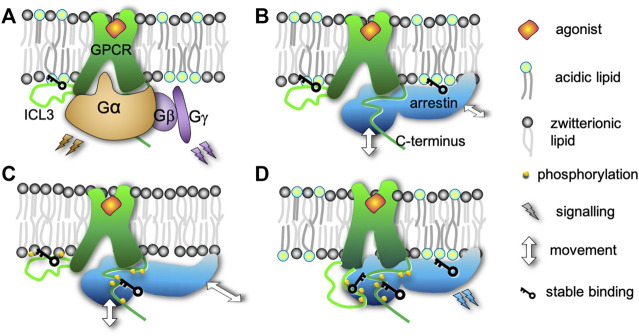
Effects of the interplay of acidic lipids and receptor phosphorylation on the complex of adrenaline-activated β_2_-adrenergic receptor with β-arrestin2. **(A)** In the complex of the receptor with a G protein the interactions between ICL3 and acidic lipids cause wide opening of the receptor cytosolic binding pocket enabling binding of the α subunit of the G protein. **(B)** Similarly to A, the cytosolic binding pocket is widely open if the receptor is not phosphorylated and embedded in a membrane with acidic lipids. Consequently, arrestin is not inserted as deeply into the receptor core. However, the acidic lipids still attach arrestin to the membrane surface. **(C)** If the receptor is phosphorylated but embedded in a membrane lacking acidic lipids, the ICL3 binds to the membrane and widely opens the cytosolic pocket. In contrast to B, the lack of acidic lipids causes looser interactions between arrestin and the membrane increasing the rotational flexibility of arrestin relative to the receptor. The phosphorylated C-terminus attaches firmly to the arrestin. **(D)** The presence of both phosphorylation and acidic lipids causes the complex to be the most compact by three mechanisms. (i) ICL3 interacts with the arrestin instead of the membrane. The lack of ICL3 interactions with the membrane makes the cytosolic binding pocket smaller and enables tighter interaction with arrestin. (ii) The phosphorylated C-terminus binds to the N-lobe of arrestin and (iii) acidic lipids attach arrestin to the membrane surface. Thus, the conformation and dynamics of binding are modulated, which is presumed to steer different intracellular signaling pathways.

## Conclusion

Our extensive atomistic MD simulations have revealed that the interplay of native acidic lipids and β_2_AR phosphorylation plays multifold roles in formation of the (p)β_2_AR*/β-arrestin2 complex (Figure 5). At first, acidic lipids repel the phosphorylated C-terminus of the receptor from the membrane surface, thus exposing the C-terminus to the cytosol for arrestin binding. Moreover, liberation of ICL3 from the negatively charged membrane surface by phosphorylation 1) enables a tighter interaction of the receptor core with arrestin’s fingerloop by allowing an inward movement of TM6 and 2) facilitates the interactions of arrestin with ICL3. A lack of acidic lipids, on the other hand, prevents arrestin from inserting deeply into the receptor core by attraction of the phosphorylated ICL3 to the membrane surface. The ICL3-membrane interactions increase the size of the cytosolic binding pocket of the receptor analogous to the nonphosphorylated receptor in the negatively charged membrane. A lack of acidic lipids also markedly enhances the rotational freedom of arrestin in contrast to the rather singular orientation of arrestin in similarly loose complexes with a nonphosphorylated receptor. Thus, the increased rotational flexibility results from the lack of attachment of the highly positively charged C-lobe of arrestin to the membrane surface via acidic DOPG lipids, which substitute the experimentally observed binding of phosphatidylinositols (Gaidarov et al., 1999; Milano et al., 2002; Milano et al., 2006).

For the first time, our MD simulations at coarse-grained level capture the process of pβ_2_AR*/βarr2 complex formation (shown in Supplementary Video S1), where the self-assembled complexes closely resemble the modelled pβ_2_AR*/βarr2 complexes after atomistic equilibration, as well as the rhodopsin/arrestin-1 X-Ray structure (Zhou et al., 2016) and the chimeric β_1_ARpp/βarr1 (Lee et al., 2020) and M_2_Rpp/βarr1 (Staus et al., 2020) cryoEM structures.

Further on, our simulations unravel that the experimentally observed reduction of the C-terminus flexibility upon phosphorylation result from interactions of the C-terminus with the receptor and not the originally suggested attachment of the C-terminus to the membrane (Shiraishi et al., 2018). The binding probability of ICL3 to the membrane surface as obtained from our simulations is further corroborated by the recently published hydrogen-deuterium exchange experiments of β_2_AR (Zhang et al., 2010).

Additionally, we shed light on individual interactions between pβ_2_AR* and βarr2. Apart from the expected interactions of arrestin’s fingerloop with the receptor core as well as of the phosphorylated C-terminus of the receptor with arrestin, we reveal that the pβ_2_AR*/βarr2 complex is stabilized by two salt bridges between arrestin and TM3 and TM5 of the receptor, respectively. Interestingly, F139, which is located in the helical ICL2 of the receptor and inserts into the crevice between arrestin’s N- and C-lobe, repels a number of arrestin residues. Given the facts that 1) the C-edge of arrestin is inserted deeply into the membrane, 2) DOPG lipids attach the positively charged C-lobe to the membrane surface and 3) the fingerloop on the N-lobe is attracted to the cytosolic binding pocket of the receptor, F139, acting on the interface of both lobes, restricts the insertion depth of arrestin into the receptor and likely also arrestin’s orientation.

The results presented here build a basis for further investigations of the regulation mechanisms of extracellular signal transmission into the cell by post-translational modifications and membrane composition. Particularly, our simulations have pinpointed residues important for the stabilization of the pβ_2_AR*/βarr2 complex. This knowledge is of pharmacological importance because currently only the activation state of the receptor is targeted by pharmaceuticals as opposed to other modulators of GPCR activity. Arrestin, on the other hand, competes with G proteins for receptor binding and causes receptor desensitization, thus opening new pathways for pharmacological intervention.

## Methods

### Structure Preparation

The structure of β_2_AR* (comprising residues 29–362) with bound adrenaline was based on the crystal structure 3SN6 (Rasmussen et al., 2011). The inactive receptor was modelled based on the crystal structure 3NY8 (Wacker et al., 2010) with the inverse agonist ICI 118551. For reasons of compatibility with ongoing research in our group we used β_2_AR with following mutations: three reactive cysteines were removed (C77V, C327S, C341L) and two methionine-to-threonine mutations that boost expression introduced (M96T, M98T). The glycosylation site is also removed (N187E). None of these mutations are located in the ICL3 or in the C-terminus. E122, localized deep in the hydrophobic membrane core, was protonated. The phosphorylation pattern of pβ_2_AR* including S246, S261, S262 on the intracellular loop 3 and S345, S346, S355, S356, and T360 on the C-terminus originates from Komolov et al. (2017). All phosphates in the phosphorylation sites were doubly negatively charged. ICL3 and C-terminus were modelled as loops using Modeler9.19 (Webb and Sali, 2016) and diverse models were chosen in which ICL3 and C-terminus are extended in the solution and not overlapping with any protein or the membrane.

The C-terminus binding orientation on arrestin in (p)β_2_AR*/βarr2 complexes was modeled based on the vasopressin receptor 2 (V2R) structure in 4JQI (Shukla et al., 2013). Thereby the two main phosphorylation sites S355 and S356 (Tran et al., 2004; Trester-Zedlitz et al., 2005) align with E356 and S357 of the V2R C-terminus (thus binding to the positively charged residues K12, K139, R166, K161). It is interesting to note that the C-terminal residues H406-T416 of the neurotensin receptor 1 (N1R) in 6PWC (Yin et al., 2019) bind to β-arrestin1 analogously to the C-tail of the vasopressin receptor with possible phosphorylation sites at the same position, i.e., T407 of N1R is in the same position as S357 of V2R in β_1_ARpp/βarr1 (6TKO) (Lee et al., 2020) and S409, S410 are in the position of T359 and T360. In most (p)β_2_AR*/βarr2 complexes the cytoplasmic half of TM6 from 3SN6 was moved to the position of the TM6 in the rhodopsin/arrestin-1 structure 5DGY (Zhou et al., 2016) in order to assure tight binding of arrestin’s fingerloop.

Also in the sequence of the human βarr2 (2–349) the cysteines were mutated (C17S, C60V, C126S, C141L, C151V, C243V, C252V, C270S, S267C C409S). The 5TV1 (Chen et al., 2017) structure was overlaid on the arrestin-1 of 5DGY (Zhou et al., 2016) or on structures after reverse transformation (backmapping) of self-assembled coarse-grained structures (the self-assembly process is exemplarily shown in Supplementary Video S1). Thereby, the structure of the fingerloop was taken from the structure it was fitted to, i.e., 5DGY, or backmapped structures (see below for details). Alternatively, β-arrestin1 in the 6TKO complex (Lee et al., 2020) was mutated to β-arrestin2.

A simple membrane mimic containing phosphatidylcholine lipids as the main constituents of the plasma membrane, phosphatidylglycerol lipids carrying a net negative charge, and cholesterol, which is known to be important for physiological function of β_2_AR (Xiang et al., 2002) was used. In detail, DOPC:DOPG:cholesterol were mixed in 54:36:10 molar ratio and symmetrically distributed over the membrane leaflets. In the simulations performed in the “neutral membrane”, DOPG was substituted for 1,2-dioleoyl-sn-glycero-3-phosphoethanolamine (DOPE).

### Simulation Setup

The setup of the individual simulations followed our well established workflow (Pluhackova et al., 2013). In detail, the proteins and their complexes were energy minimized atomistically *in vacuo* and then converted to a coarse-grained (CG) representation of Martini2.2p (Yesylevskyy et al., 2010; de Jong et al., 2013; de Jong et al., 2016) using the tool *martinize* (de Jong et al., 2013) and energy minimized *in vacuo*. Next, membrane and solvent were built using the tool *insane* (Wassenaar et al., 2015). These CG structures were energy minimized and relaxed by a series of short equilibration simulations keeping the proteins position restrained (500 ps with 10 fs timestep and 1 ns with 20 fs timestep). Further on, equilibration simulations at CG resolution were performed using 20 fs time step and the final structures were converted back to atomistic resolution using the tool *backward* (Wassenaar et al., 2014). On these structures the original energy minimized atomistic protein structures were fitted, the overlapping water molecules and ions in a direct vicinity removed and the system was energy minimized in three steps. In the first energy minimization of 5,000 steps the proteins and the ligands (adrenaline or ICI) were kept frozen. In the second energy minimization of 5,000 steps only the protein backbone was frozen and in the third energy minimization of 5,000 steps all atoms were allowed to move freely. The systems were then equilibrated in two simulations with position restraints on the protein. In the first equilibration simulation lasting 20 ns all heavy atoms of proteins and ligands were position restrained, while in the second simulation (lasting 10 ns) only the protein backbone atoms were position restrained. The following production run simulations lasted 1–2 μs. A list of all production run simulations is shown in Supplementary Table S1. The first 500 ns of each simulation was excluded from analysis for equilibration purposes, the remaining simulation time was split into 500 ns time intervals for analysis.

### Simulation Conditions

All molecular dynamics simulations were performed at 310 K using semiisotropic pressure coupling to 1 bar in GROMACS 2018 (Abraham et al., 2015). At all-atom resolution the CHARMM36 force field was used for lipids (Klauda et al., 2010) and cholesterol (Lim et al., 2012), proteins were described by CHARMM36m (Huang et al., 2017). The corresponding force field parameters for adrenaline and ICI118551 were generated by CHARMM-Gui (Jo et al., 2008) and water was represented by the TIP4p water model (Jorgensen and Madura 1985).

Because of the requirement to describe properly the electrostatic interactions (Pluhackova et al., 2015) the polarizable variant of the Martini force field version 2 was utilized in the coarse-grained simulations (Marrink et al., 2007; Yesylevskyy et al., 2010; de Jong et al., 2013). The parameters for phosphorylated serine and threonine were developed in-house and are summarized in the Supplementary Data Sheet S1.

The simulation parameters at all-atom resolutions followed our well tested setup for the CHARMM36 force field (Pluhackova et al., 2016; Sandoval-Perez et al., 2017). In coarse-grained simulations the recommendation of de Jong et al. were applied (de Jong et al., 2016). For details, see Supplementary Data Sheet S1, section Simulation conditions.

### Replica Exchange Solute Tempering MD Simulations

In order to achieve sufficient sampling of the membrane binding of the unstructured ICL3 and C-terminus, the following adaptation of the replica exchange solute tempering (REST) (Liu et al., 2005) method was applied: the receptor, adrenaline, membrane and solvent (water and ions) were each independently coupled to a temperature bath. While the protein was heated to 310, 360, 410, 460, and 510 K, the other components were kept at 310, 310.1, 310.2, 310.3, and 310.4 K thus assuring for stable area per lipid of the bilayer enabling high probability of replica exchange amounting to 70–80%. Replica exchange was attempted every 500 steps, i.e., each picosecond. The secondary structure of the transmembrane domain of the receptor was stabilized by distance restraints among Cα atoms within 0.9 nm. The distance range around the actual distance for generating distance restraints amounted to 25% of the bond length and the distance between upper bound for distance restraints, and the distance at which the force becomes constant was 0.5 nm (-disre_up2). The REST MD simulations were run for 200 ns, the convergence of the membrane binding probabilities was monitored over 50 ns intervals. For analysis only the last two 50-ns intervals, i.e., between 100 and 200 ns of the replica at 310 K, were used. For each membrane and receptor type three independent REST MD simulations were performed initialized from different models of the ICL3 and the C-terminus.

All plots were generated in R version 3.5.4 (Core Team 2020) and molecular visualizations were prepared in PyMOL version 2.5 (Schrödinger 2020).

## Data Availability

The original contributions presented in the study are included in the article/Supplementary Material, further inquiries can be directed to the corresponding author.
