# Supplementary material for: Lipids and Phosphorylation Conjointly Modulate Complex Formation of β2-Adrenergic Receptor and β-arrestin2
Source: Front Cell Dev Biol. 2021 Dec 23;9:807913. doi: 10.3389/fcell.2021.807913 (PMC8733679; doi:10.3389/fcell.2021.807913)
Supplement: Supplementary file 1 [file DataSheet1.pdf]

Supplementary Material: Lipids and phosphorylation conjointly modulate complex formation of β_2-adrenergic receptor and β-arrestin2

Kristyna Pluhackova*, Florian M. Wilhelm, Daniel J. Müller

Department of Biosystems Science and Engineering, Eidgenössische Technische Hochschule (ETH) Zurich, Basel, Switzerland

* Correspondence: kristyna.pluhackova@bsse.ethz.ch

https://www.frontiersin.org/articles/10.3389/fcell.2021.807913/full

**Simulation system**	**Simulation type and length**	**Notes**
pβ_2AR* in CM	3 x REST (5 x 200 ns)<br>4 x 1 µs, 1.5 µs, 2 µs MD	
β_2AR* in CM	3 x REST (5 x 200 ns)<br>5 x 1 µs	
pβ_2AR* in NM	3 x REST (5 x 200 ns)	
β_2AR* in NM	3 x REST (5 x 200 ns)	
β_2AR in CM	3 x REST (5 x 200 ns)	
pβ_2AR in CM	3 x REST (5 x 200 ns)	
β_2AR*/G_s protein in CM	3 x REST (5 x 200 ns)<br>3 x 1 µs MD	
pβ_2AR*/βarr2 in CM	2 x 1 µs, 5 x 2 µs	
β_2AR*/βarr2 in CM	5 x 1 µs, 1 x 2 µs	In one 1-µs simulation arrestin unbound from the receptor core → excluded from the analysis
pβ_2AR*/βarr2 in NM	5 x 1 µs, 4 x 2 µs	In two 1-µs simulations arrestin unbound from the receptor core → excluded from the analysis
27 pSer in solution	0.5 µs	for Martini parametrization
27 pThr in solution	0.5 µs	for Martini parametrization
**Total simulation time**	**69.5 µs**	

Supplementary Table 1: List of all-atom simulations. Charged membranes (CM) are composed of DOPC, DOPG, and cholesterol, and neutral membranes (NM) are composed of DOPC, DOPE, and cholesterol. pβ_2AR* indicates phosphorylated receptor activated by adrenaline, β_2AR* denotes nonphosphorylated receptor activated by adrenaline, β_2AR and pβ_2AR are nonphosphorylated or phosphorylated receptors, respectively, with bound inverse agonist ICI 118551.

Simulation conditions

In coarse-grained (CG) simulations, the simulation input parameters for the polarizable Martini2.2p[1] force field, including the PW water model[2], were used as recommended for GROMACS5 and GROMACS2016[3]. In detail, the temperature of 310 K was controlled by the velocity rescale thermostat[4]. The pressure was coupled in a semiisotropic manner to 1 bar by the Parrinello-Rahman barostat[5, 6], using 12 ps time constant and compressibility of 3e-4 bar^{-1}. The integration step amounted to 20 fs. The Verlet cut-off scheme[7] was used for neighbor search and the van der Waals forces were switched to zero at 1.1 nm over the full interaction length by the Potential-shift-Verlet modifier. PME[8] was utilized to describe the electrostatic interactions above the 1.1-nm cutoff, and the relative permittivity, ε_r, was 2.5.

In the atomistic simulations performed with the CHARMM36m force field[9-11], a 2-fs integration step was used. The Verlet cut-off scheme[7] accomplished neighbor search with buffer tolerance of 0.005 kJ/mol/ps per atom. PME[8] was utilized to describe electrostatics above a 1.2-nm cutoff and the Potential-switch function switched the 12-6 Lennard-Jones potential to zero between 0.8 and 1.2 nm. The temperature of 310 K was controlled by the Nosé-Hoover[12] thermostat with the time constant of 0.5 ps. Parrinello-Rahman barostat[5, 6] with the time constant of 5 ps controlled the pressure of 1 bar in a semiisotropic manner using the compressibility of 4.5e-5 bar^{-1}. The center of mass motion of the system was linearly removed every 100 steps.

Parametrization of phosphorylated amino acids within the framework of Martini2.2 and Martini2.2p.

As atomistic reference data, 500-ns-long all-atom simulations using CHARMM36 force field of 27 individual phosphorylated residues including a backbone with neutral termini were performed separately for phosphoserine and phosphothreonine, each carrying two negative charges on the phosphate. The phosphorylated residues were solvated (1:100) by TIP3p water and neutralized by Na+ counterions.

Residue	Bead name	Bead type	Charge
SP2 phosphoserine	BB	P2*	0
	SC1	P1	0
	PO4	Qa	-2

Supplementary Table 2: Phosphoserine bead types, names and charges in Martini2.2 representation. *The type of the backbone bead (BB) depends on the secondary structure of the amino acid.

Residue	Bonded parameter	Involved beads	Equilibrium value	Force constant [kJ/mol/nm^2]
SP2 phosphoserine	bond	BB-SC1	0.255 [nm]	7500
	bond	SC1-PO4	0.225 [nm]	5000
	angle	BB-SC1-PO4	90°	50

Supplementary Table 3: Phosphoserine bonded parameters in Martini2.2 representation.

Residue	Parameter	Involved beads	AA simulated value	CG simulated value
SP2 phosphoserine	bond	BB-SC1	0.267 nm	0.274 nm
	bond	SC1-PO4	0.229 nm	0.251 nm
	angle	BB-SC1-PO4	127°	133°

Supplementary Table 4: Comparison of phosphoserine's structure in CG simulations and in coarse-grained atomistic simulation.

Residue	Bead name	Bead type	Charge
THP2 phosphothreonine	BB	P2*	0
	SC1	P1	0
	PO4	Qa	-2

Supplementary Table 5: Phosphothreonine bead types, names and charges in Martini2.2 representation. *The type of the backbone bead (BB) depends on the secondary structure of the amino acid.

Residue	Bonded parameter	Involved beads	Equilibrium value	Force constant [kJ/mol/nm^2]
THP2 phosphothreonine	bond	BB-SC1	0.240 [nm]	7500
	bond	SC1-PO4	0.235 [nm]	5500
	angle	BB-SC1-PO4	50°	70

Supplementary Table 6: Phosphothreonine bonded parameters in Martini2.2 representation.

Residue	Bonded parameter	Involved beads	AA simulated value	CG simulated value
THP2 phosphothreonine	bond	BB-SC1	0.271 nm	0.275 nm
	bond	SC1-PO4	0.280 nm	0.285 nm
	angle	BB-SC1-PO4	108°	111°

Supplementary Table 7: Comparison of phosphoserine's structure in CG simulations and in coarse-grained atomistic simulation.

GROMACS itp files for phosphoserine and phosphothreonine for Martini2.2 force field

```
[ moleculetype ]
Phosphoserine        1
[ atoms ]
   1    P2    1   SP2   BB     1  0.0000
   2    P1    1   SP2   SC1    2  0.0000
   3    Qa    1   SP2   PO4    3 -2.0000
[ bonds ]
   1     2     1   0.255  7500
   2     3     1   0.225  5000
[ angles ]
  1  2  3      2       90.0   50.0
```

```
[ moleculetype ]
Phosphothreonine        1
[ atoms ]
   1    P2    1   THP2   BB     1  0.0000
   2    P1    1   THP2   SC1    2  0.0000
   3    Qa    1   THP2   PO4    3 -2.0000
[ bonds ]
   1     2     1   0.240  7500
   2     3     1   0.235  5500
[ angles ]
  1  2  3      2       50.0   70.0
```

In case of Martini2.2p, the bonded parameters were kept the same, but the charge and mass were moved onto two dummy beads.

GROMACS itp files for phosphoserine and phosphothreonine for Martini2.2p force field

```
[ moleculetype ]
Phosphoserine        1
[ atoms ]
   1   P2    1   SP2   BB      1      0.0000      72.0
   2   P1    1   SP2   SC1     2      0.0000      72.0
   3   Qa    1   SP2   PO4     3      0.0000      0.0
   4   D     1   SP2   SCN     4      -1.0000     36.0
   5   D     1   SP2   SCN     5      -1.0000     36.0
[ bonds ]
   1    2    1   0.255  7500
   2    3    1   0.225  5000
[ constraints ]
   4    5    1   0.280
[ angles ]
  1  2  3     2      90.0   50.0
[ virtual_sites2 ]
  3  4  5     1      0.500


[ moleculetype ]
Phosphothreonine        1
[ atoms ]
   1   P2    1   THP2   BB          1      0.0000      72.0
   2   P1    1   THP2   SC1         2      0.0000      72.0
   3   Qa    1   THP2   PO4         3      0.0000      0.0
   4   D     1   THP2   SCN         4      -1.0000     36.0
   5   D     1   THP2   SCN         5      -1.0000     36.0
[ bonds ]
   1    2    1   0.240  7500
   2    3    1   0.235  5500
[ constraints ]
   4    5    1      0.280
[ angles ]
  1  2  3     2      50.0   70.0
[ virtual_sites2 ]
  3  4  5     1      0.500
```

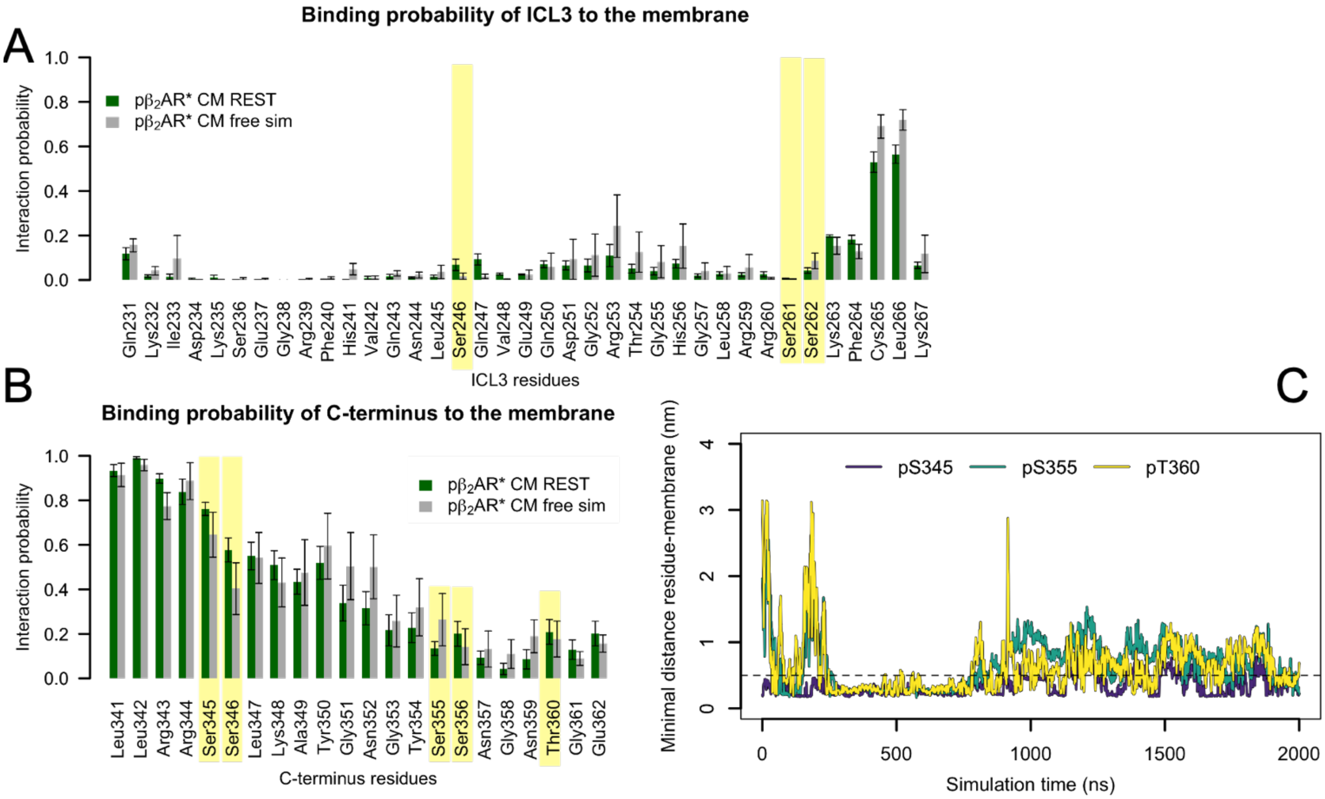

Supplementary Figure 1: Comparison of membrane binding probabilities of ICL3 and C-terminus from pβ$_2$AR* as obtained from REST and free MD simulations. A. Interaction probabilities of ICL3 residues with the membrane. **B.** Interaction probabilities of C-terminal residues to the membrane. Yellow background indicates residues that are phosphorylated in the respective data sets. The interaction patterns obtained by the two methods are almost identical. The estimated error is smaller in REST MD simulations. Moreover, REST saves computational resources: the REST MD simulations performed here amounted to 3 μs simulation time, while the results from the free MD simulations were obtained from six independent MD simulations lasting 7.5 μs in total. The error bars denote standard error of the mean obtained over six 50-ns intervals from REST MD simulations (considering only 100-200-ns time intervals of three independent REST MD simulations) and from nine 500-ns intervals of the free MD simulations (after excluding first 500 ns of each simulation for equilibration purposes). **C.** Exemplary minimal distances between three phosphorylated residues and a negatively charged membrane surface over the course of a 2-μs long MD simulation. The horizontal line depicts the minimal distance of 0.5 nm used as an interaction criterium in A and B. Both persistent binding events, lasting several hundreds of ns as well as temporary binding events are observed.

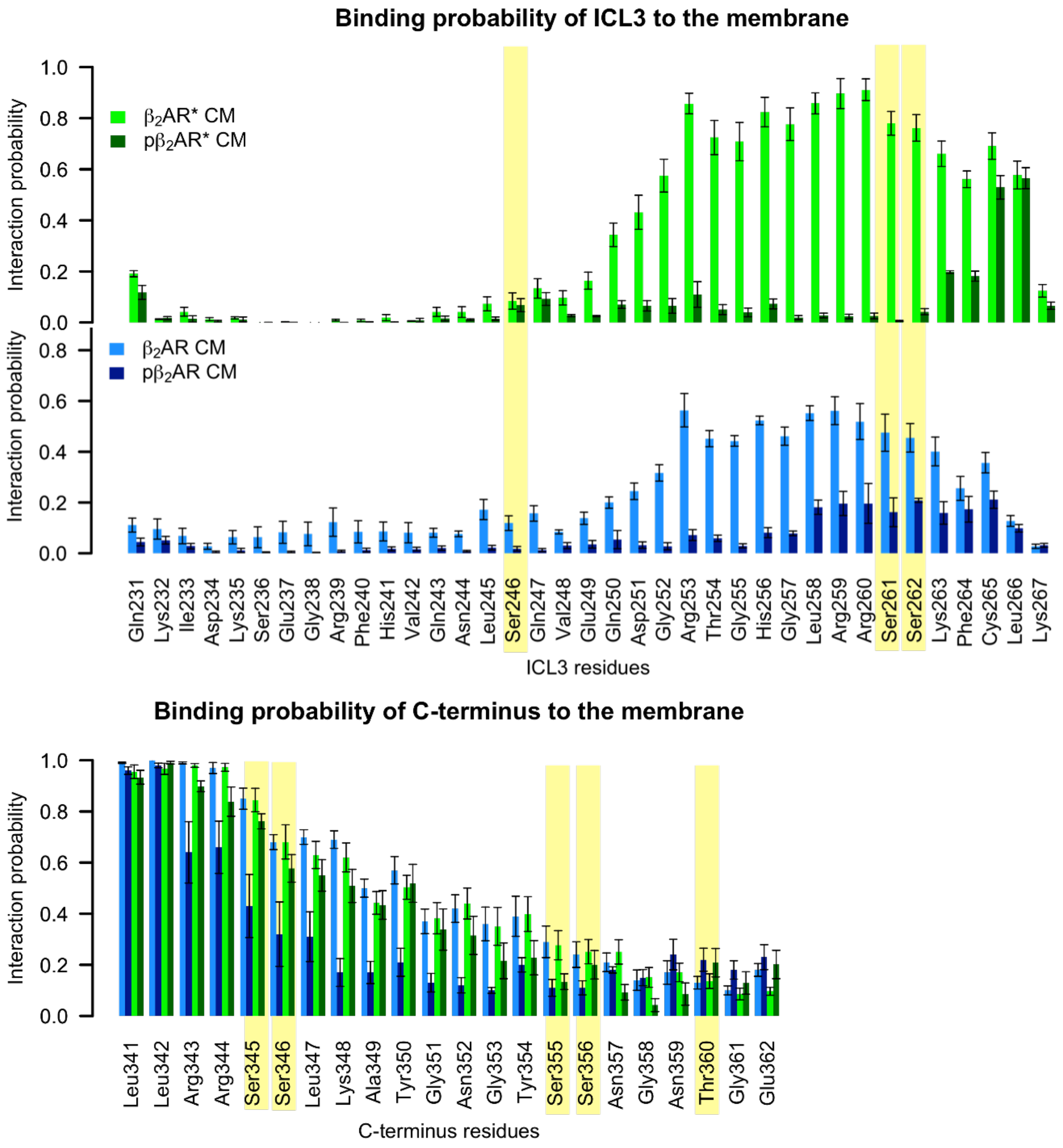

Supplementary Figure 2: Comparison of membrane binding probabilities of ICL3 and C-terminus from the active and inactive β_2AR prior and post phosphorylation. β_2AR with bound inverse agonist ICI 118551 (blue shades) shows similar membrane binding patterns of ICL3 (top) and C-terminus (bottom) as the receptor with bound agonist adrenaline, β_2AR* (green shades). However, the binding probability of the TM6-proximal half of ICL3 is reduced by almost 50% in the inactive nonphosphorylated state. Moreover, after phosphorylation, residues Leu258-Ser262 show slightly higher and residues Cys265-Leu266 lower membrane binding probabilities than in the active state. The phosphorylated inactive receptor exhibits decreased membrane binding probabilities for C-terminus residues Arg343-Ser356 as compared to the other complexes. The error bars denote standard error of the mean obtained over six 50-ns intervals from REST MD simulations (considering only 100-200-ns time intervals of three independent REST MD simulations). Yellow background indicates residues that are phosphorylated in the respective data sets.

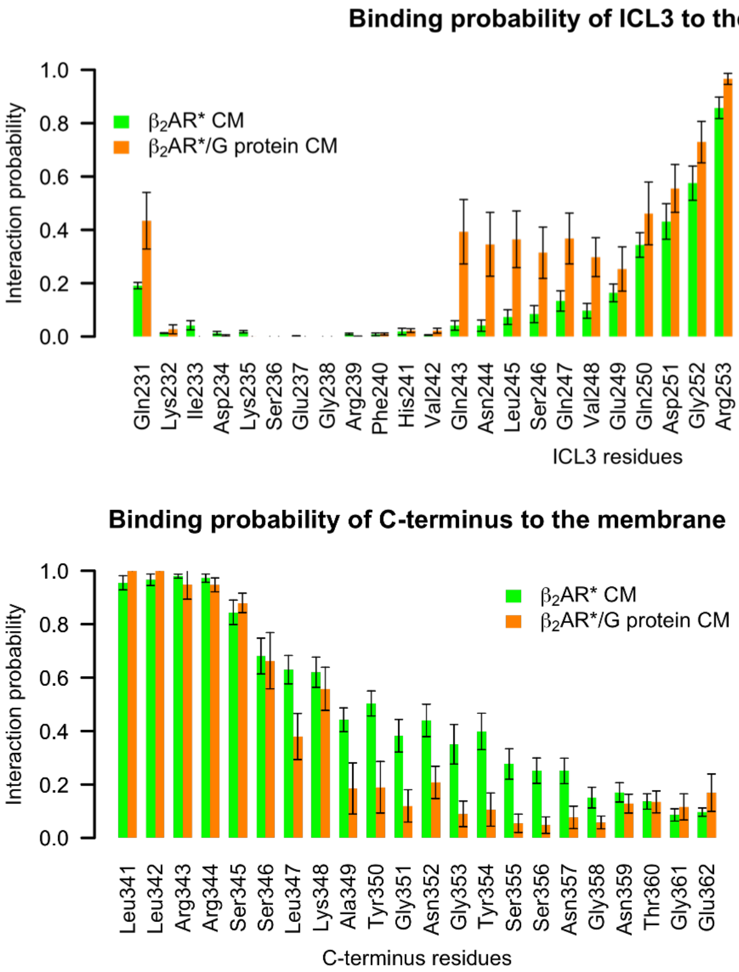

Supplementary Figure 3: Comparison of membrane binding probabilities of ICL3 (top) and C-terminus (bottom) of the adrenaline-bound β$_2$AR* without any intracellular binding partner (in green) and in complex with the trimeric G$_s$ protein complex (in orange). The error bars denote standard error of the mean obtained over six 50-ns intervals from REST MD simulations (considering only 100-200-ns time intervals of three independent REST MD simulations).

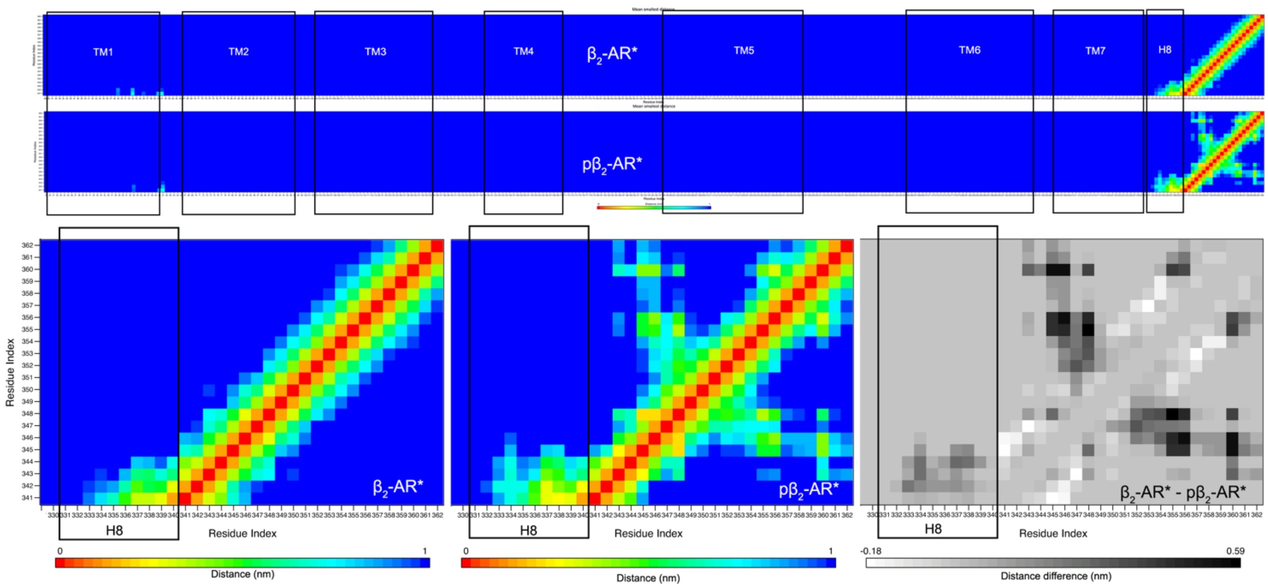

Supplementary Figure 4: Interactions of receptor C-terminus residues with all receptor residues evaluated as average distances between C-terminus residues and receptor residues over the simulation time and all simulations of a given type. Top two rows: C-terminus (rightmost) interacts with itself and with helix 8. The seemingly close contacts with TM1 result from their neighborhood in the protein structure and do not result from direct interactions. **Bottom row**: detailed view on the interactions of helix 8 and C-terminus in the nonphosphorylated state (β_2AR*, left), phosphorylated state (pβ_2AR*, middle), and their difference (β_2AR*-pβ_2AR*, right). The rightmost map is colored white to black (white colored residues are further apart in pβ_2AR* than in β_2AR* and black colored residues are closer in pβ_2AR* than in β_2AR*). All other maps are rainbow colored red to blue, for distances from 0 to 1 nm and larger. Data obtained from 7 pβ_2AR* CM and 5 β_2AR* CM independent MD simulations, first 500 ns were excluded from the analysis for equilibration purposes.

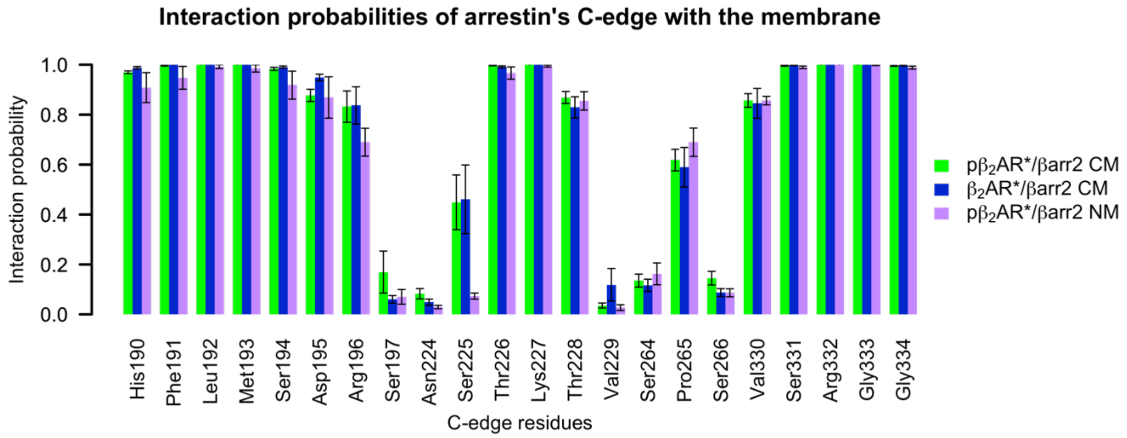

Supplementary Figure 5: Interaction probabilities of arrestin's C-edge residues to the membrane in different receptor/arrestin complexes. Error bars represent standard error of the mean obtained from 17 (pβ_2AR*/βarr2 CM), 15 (pβ_2AR*/βarr2 NM), and 7 (β_2AR*/βarr2 CM) 500-ns intervals of free MD simulations. First 500 ns of each simulation were omitted for equilibration purposes.

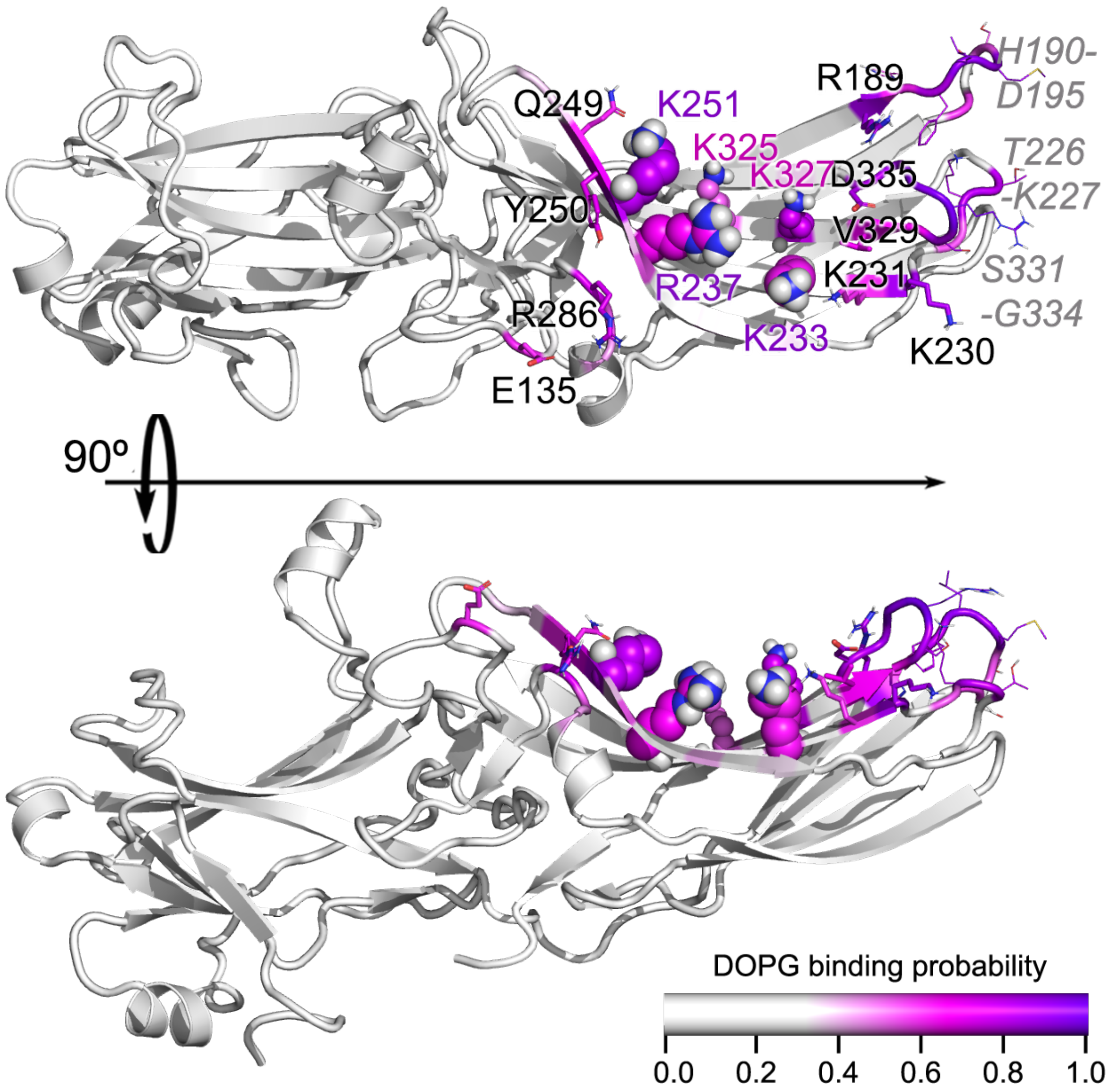

Supplementary Figure 6: Binding probability of DOPG lipids to the individual residues of arrestin in the pβ$_2$AR*/βarr2 complex. Top view from the membrane**, bottom,** view along the membrane plane. The residues K251, R237 and K233, which were identified to bind phosphatidylinositol lipids[13], are shown as large spheres and labeled in purple. The same residues and additionally the residues K327 and K325 (shown as small spheres and labeled in magenta) were experimentally shown to bind hexakisphosphate[14]. Residues at the C-edge which insert into the bilayer are shown as lines and labeled in grey. Additional residues identified here to attract DOPG are shown as sticks and labeled in black. The binding probability of 1 means that at least one DOPG lipid is located within 0.5 nm (a distance reflecting direct hydrogen bonding and hydrogen bonds mediated by a single water molecule) to the given residue for 100% of the analyzed simulation time (first 500 ns were removed from all simulations for equilibration purposes, N=7).

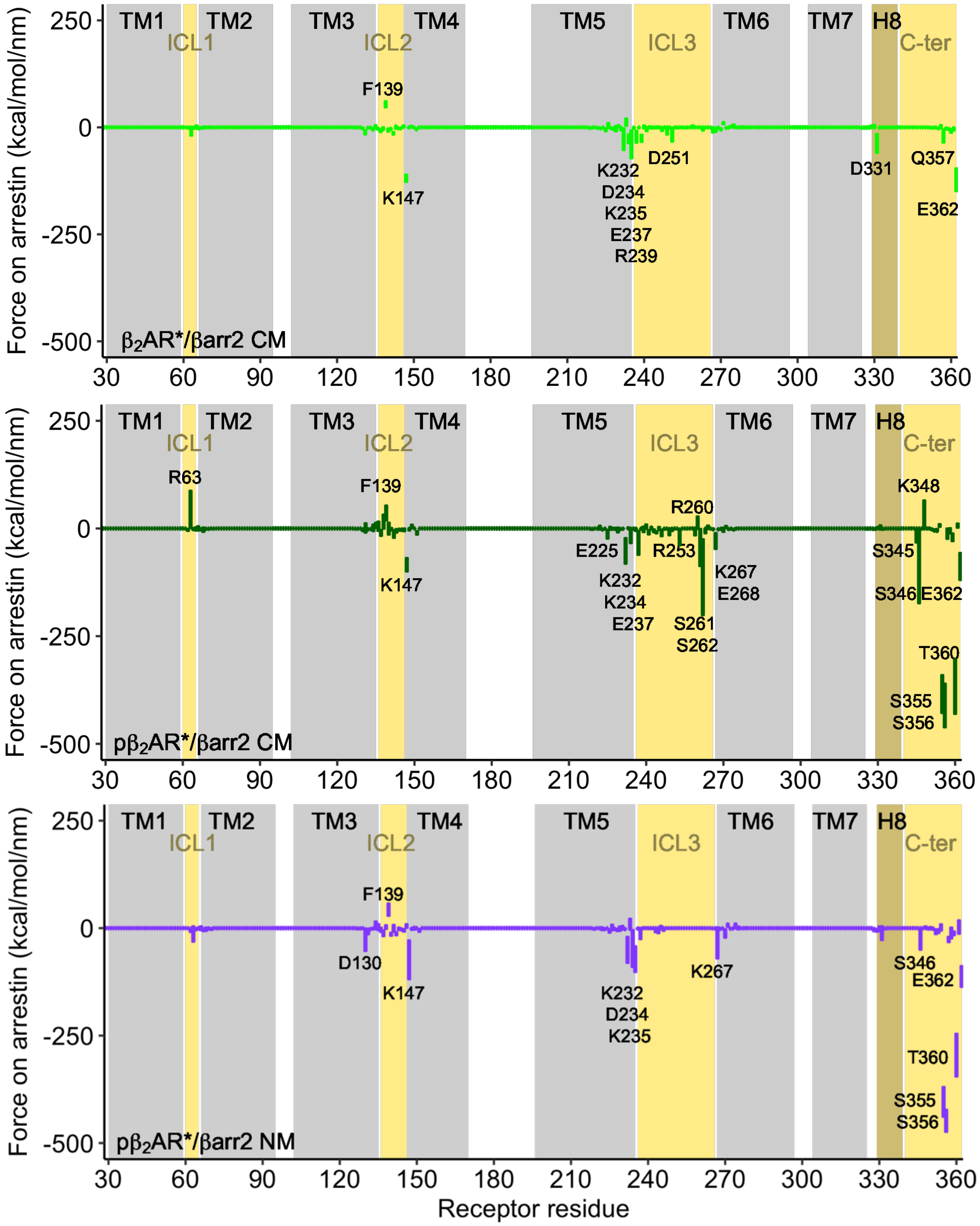

Supplementary Figure 7: Simplified boxplot of average forces receptor residues exert on arrestin. Positive values denote repulsive forces and negative values denote attractive forces. Top β$_2$AR/βarr2 in the charged membrane (CM) (6 simulations, seven 500-ns analysis intervals), middle pβ$_2$AR*/βarr2 in CM (7 simulations, seventeen 500-ns analysis intervals), bottom pβ$_2$AR*/βarr2 in the neutral membrane (NM) (7 simulations, fifteen 500-ns analysis intervals). First 500 ns of each MD simulation were excluded for equilibration purposes. The boxplot is simplified for clarity by omission of the mean value and the outliers as well as the whiskers.

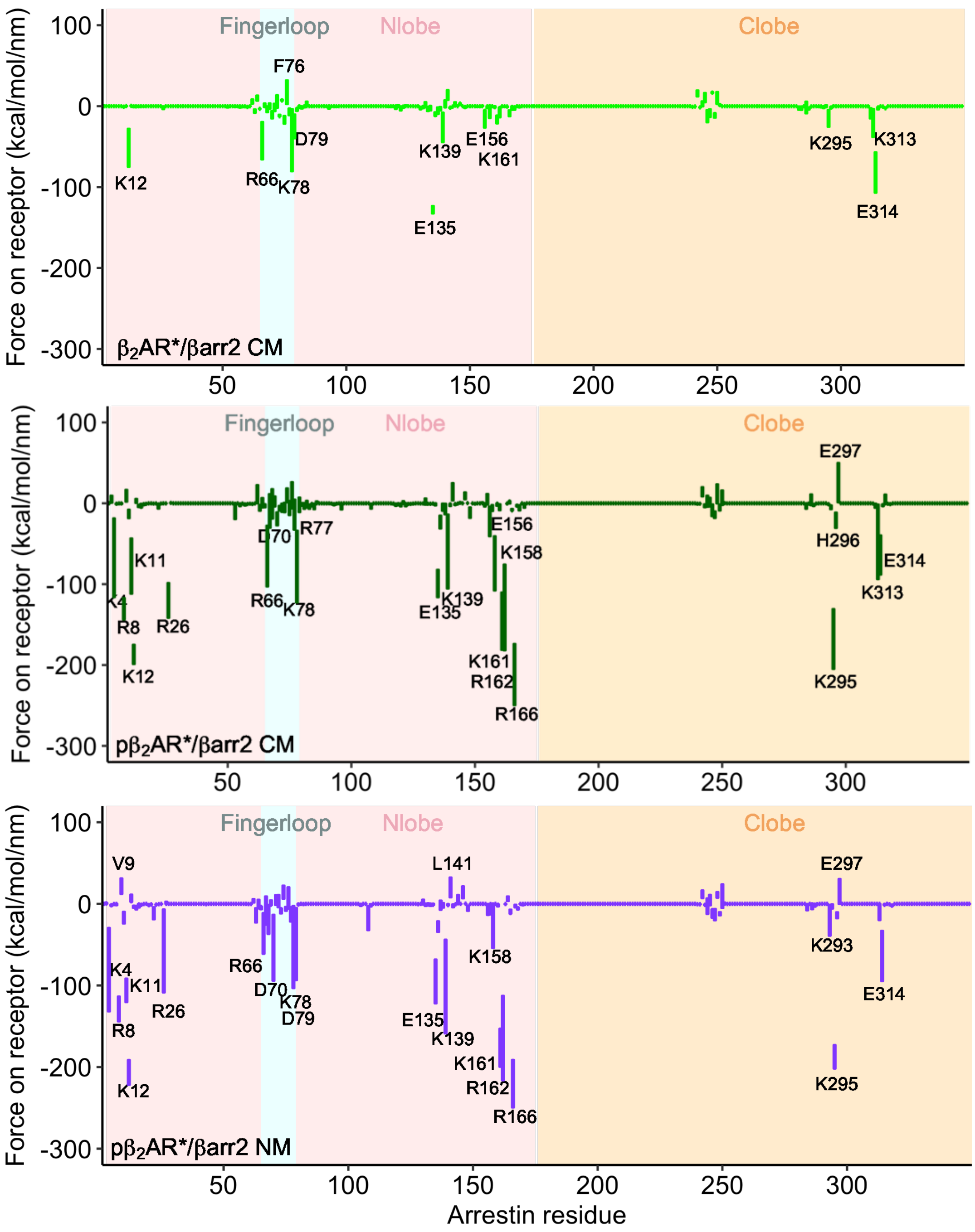

Supplementary Figure 8: Simplified boxplot of average forces arrestin residues exert on the receptor. Positive values denote repulsive forces and negative values denote attractive forces. Top β2AR*/βarr2 in CM (6 simulations, seven 500-ns analysis intervals), middle pβ2AR*/βarr2 in CM (7 simulations, seventeen 500-ns analysis intervals), bottom pβ2AR*/βarr2 in NM (7 simulations, fifteen 500-ns analysis intervals). First 500 ns of each MD simulation were excluded for equilibration purposes.

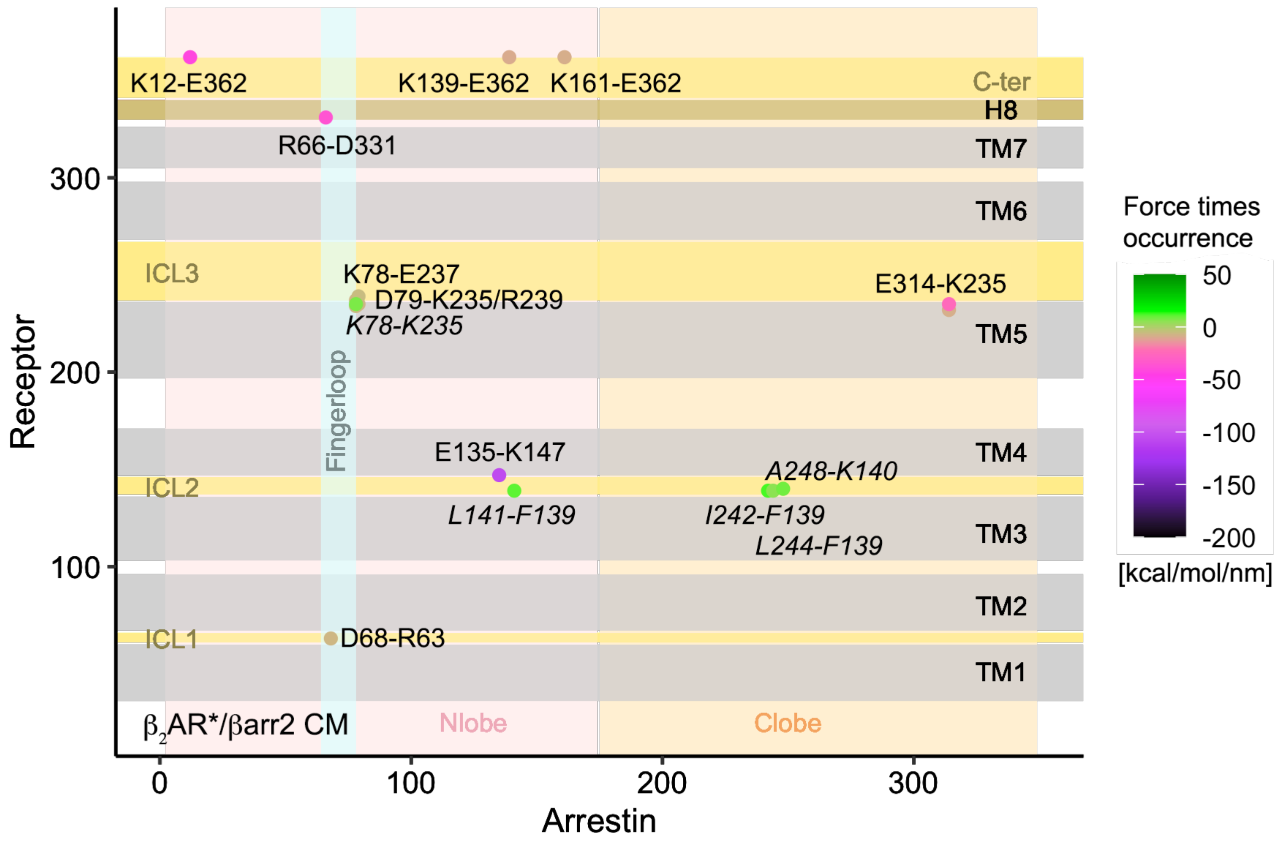

Supplementary Figure 9: Interacting pairs of arrestin and receptor residues in the β$_2$AR*/βarr2 complex in the charged membrane. As a classification criterium the average force between each two residues was multiplied by the probability to form a pair stronger than 10 kcal/mol/nm. Positive values denote repulsive forces, negative values denote attractive forces. The labels of the most significant repulsive pairs are in italics and those of the attractive pairs in a regular typeface. For values of forces and occurrence of all residue pairs see **Supplementary File Data Sheet 2.xlsx.** The data were collected over seven 500-ns simulation intervals obtained from six independent MD simulations, first 500 ns of each simulation were excluded for equilibration purposes.

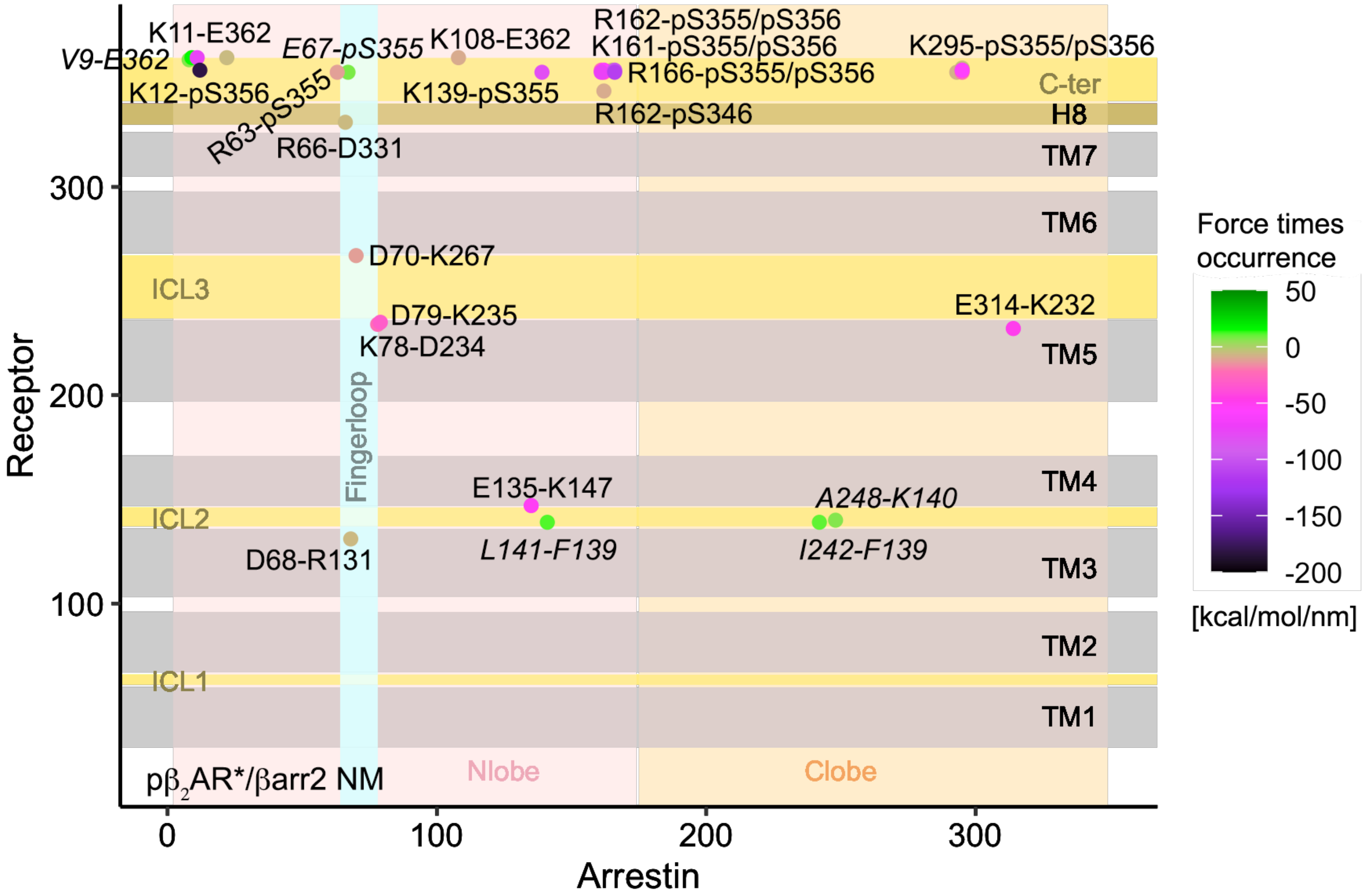

Supplementary Figure 10: Interacting pairs between arrestin and receptor residues in the pβ₂AR/βarr2 complex in the neutral membrane. As a classification criterium the average force between each two residues was multiplied by the probability to form a pair stronger than 10 kcal/mol/nm. Positive values denote repulsive forces, negative values denote attractive forces. The labels of the most significant repulsive pairs are in italics and those of the attractive pairs in a regular typeface. For values of forces and occurrence of all residue pairs see **Supplementary File Data Sheet 2.xlsx**. The data were collected over fifteen 500-ns simulation intervals obtained from seven independent MD simulations, first 500 ns of each simulation were excluded for equilibration purposes.

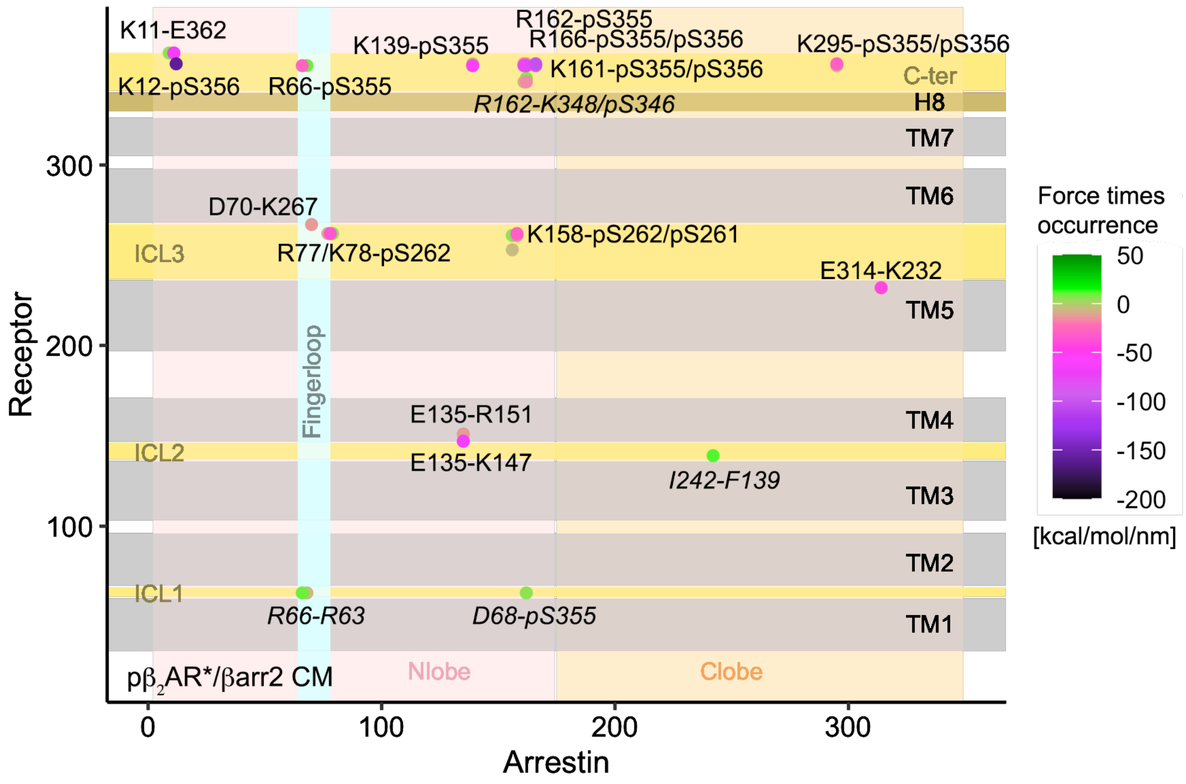

Supplementary Figure 11: Interacting pairs between arrestin and receptor residues in the pβ2AR*/βarr2 complex in the charged membrane. As a classification criterium the average force between each two residues was multiplied by the probability to form a pair stronger than 10 kcal/mol/nm. Positive values denote repulsive forces, negative values denote attractive forces. The labels of the most significant repulsive pairs are in italics and those of the attractive pairs in a regular typeface. For values of forces and occurrence of all residue pairs see **Supplementary File Data Sheet 2.xlsx.** The data were collected over seventeen 500-ns simulation intervals obtained from seven independent MD simulations, first 500 ns of each simulation were excluded for equilibration purposes.

Supplementary Figures 9-11, and the **Supplementary File Data Sheet 2.xlsx** reveal that one of the most often occurring interactions between the receptor and the arrestin is a salt bridge between E135 (localized on arrestin's middle loop) of arrestin with K147 at the cytoplasmic end of TM4 of the receptor (Figure 4C, insert VIII). This salt bridge is found in all β2AR*/βarr2 CM simulation intervals (MD simulations were split into 500 ns long intervals and the first 500 ns from each simulation were omitted for equilibration purposes), in 16 out of 17 pβ2AR*/βarr2 CM simulation intervals and in 12 out of 15 pβ2AR*/βarr2 NM intervals. On the other side of the complex, the salt bridge between E314 of arrestin and K232 at the cytoplasmic end of TM5 of the receptor (Figure 4C, insert IV) stabilizes 12 out of 17 pβ2-AR*/βarr2 CM and 13 out of 15 pβ2AR*/βarr2 NM complexes. In 4 out of 7 β2AR*/βarr2 CM simulation intervals, E314 forms a salt bridge with K235 instead.

In addition, the arrestin fingerloop plays an important role. In 4 out of 7 β2AR*/βarr2 CM intervals a salt bridge between D68 and R63 is formed, and a salt bridge R66-D331 is present in 5 out of 7

intervals. Moreover, both S75 and F76 interact in 3 out of 7 intervals with Q229. In pβ2AR*/βarr2 CM, the following interactions of arrestin fingerloop residues with the receptor were observed: E67-R63 (7/17), D68-R63 (8/17), D70-K267 (9/17) and L74-I135 (6/17), K78-pS262 (10/17), R77-pS262 (6/17), Y64-P138 (6/17). Interestingly, R66 can stretch out to pS355 (9/17). In the pβ2AR*/βarr2 NM complex, residues of the fingerloop formed the following interactions: E67-R63 (5/15), R66-D130 (7/15), D68-R131 (5/15), K78-D234 (7/15), D70-K267 (6/15), D70-K270 (6/15), R66-D331 (5/15), R64-pS355 (6/15).

In accordance with the observed preference of the phosphorylated ICL3 to interact with the neutral membrane surface, the ICL3 of pβ2AR*/βarr2 NM interacts only in 2 out of 15 simulation intervals with arrestin, while in the charged membrane pS262 interacts in 13 out of 17 simulations strongly with arrestin. In the latter case, pS261 and/or pS262 are attracted by K78 and K158, R77, R148, K161, and K313 and repelled by D79, H156 and D70 on arrestin. Moreover, pS246 is in some simulations (5/17) attracted to K313 and K267 of ICL3 builds in 9/17 cases a salt bridge with D70 on arrestin.

The nonphosphorylated C-terminus in the β2AR*/βarr2 complex interacts with K12, K139 and K161 via E362 (in 6/7, 5/7 and 5/7 intervals, respectively). In the neutral membrane, pS346 interacts in 5 of 15 intervals with arrestin's R162 and in the charged membrane in 6 out of 17 intervals. Moreover, p355, p356, p360 and C-terminal E362 are attracted by a number of arrestin residues including K4, R8, V9, F10, K11, K12, Y22, R26, R63, R66, K108, K139, K161, R162, V165, R166, K293, K295 and H296, and repelled by E297, E67, E146, and D68.

The probabilities and the average forces of the individual residue pairs are collected in the **Supplementary File Data Sheet 2.xlsx.**

The **Supplementary Movie Video 1.MP4** shows a self-assembly of the pβ2AR*/βarr2 complex at coarse-grained (CG) resolution using the Martini2.2p force-field. The ICL3 was removed from the CG model in order to avoid possible plugging of the receptor's intracellular binding pocket prior to arrestin's insertion into it. The final conformation resembles very well the complex of rhodopsin/arrestin-1[15] (shown as grey cartoon). Similar results were obtained from three independent spontaneous binding simulations starting from different orientations of arrestin relative to the receptor. The relative orientation of arrestin in the three endpoints of the 10 µs-long self-assembly simulations in terms of insertion depth d and arrestin rotation α amount to 4.05 nm and 17.3°, 3.91 nm and 22.3°, 4.27 nm and 8.3°, respectively. The position of the membrane is visualized in the background as white sticks and spheres. The pβ2AR* is shown as red ribbon, the two main phosphorylation sites on the C-terminus pS355 and pS356 are shown as yellow spheres. The arrestin is shown as blue ribbon with the fingerloop colored green.

Supplementary References:

1. D. H. de Jong, G. Singh, W. F. D. Bennett, C. Arnarez, T. A. Wassenaar, L. V. Schäfer, X. Periole, D. P. Tieleman and S. J. Marrink, *Journal of Chemical Theory and Computation*, 2013, **9**, 687-697.
2. S. O. Yesylevskyy, L. V. Schäfer, D. Sengupta and S. J. Marrink, *PLOS Computational Biology*, 2010, **6**, e1000810.
3. D. H. de Jong, S. Baoukina, H. I. Ingólfsson and S. J. Marrink, *Computer Physics Communications*, 2016, **199**, 1-7.
4. G. Bussi, D. Donadio and M. Parrinello, *The Journal of Chemical Physics*, 2007, **126**, 014101.
5. M. Parrinello and A. Rahman, *Physical Review Letters*, 1980, **45**, 1196-1199.
6. M. Parrinello and A. Rahman, *Journal of Applied Physics*, 1981, **52**, 7182-7190.
7. S. Páll and B. Hess, *Computer Physics Communications*, 2013, **184**, 2641-2650.
8. T. Darden, D. York and L. Pedersen, *The Journal of Chemical Physics*, 1993, **98**, 10089-10092.
9. J. Huang, S. Rauscher, G. Nawrocki, T. Ran, M. Feig, B. L. de Groot, H. Grubmüller and A. D. MacKerell, *Nature Methods*, 2017, **14**, 71-73.
10. J. B. Klauda, R. M. Venable, J. A. Freites, J. W. O'Connor, D. J. Tobias, C. Mondragon-Ramirez, I. Vorobyov, A. D. MacKerell and R. W. Pastor, *The Journal of Physical Chemistry B*, 2010, **114**, 7830-7843.
11. P. Bjelkmar, P. Larsson, M. A. Cuendet, B. Hess and E. Lindahl, *Journal of chemical theory and computation*, 2010, **6**, 459-466.
12. D. J. Evans and B. L. Holian, *The Journal of Chemical Physics*, 1985, **83**, 4069-4074.
13. I. Gaidarov, J. G. Krupnick, J. R. Falck, J. L. Benovic and J. H. Keen, *The EMBO Journal*, 1999, **18**, 871-881.
14. S. K. Milano, Y.-M. Kim, F. P. Stefano, J. L. Benovic and C. Brenner, *Journal of Biological Chemistry*, 2006, **281**, 9812-9823.
15. X. E. Zhou, X. Gao, A. Barty, Y. Kang, Y. He, W. Liu, A. Ishchenko, T. A. White, O. Yefanov, G. W. Han, Q. Xu, P. W. de Waal, K. M. Suino-Powell, S. Boutet, G. J. Williams, M. Wang, D. Li, M. Caffrey, H. N. Chapman, J. C. H. Spence, P. Fromme, U. Weierstall, R. C. Stevens, V. Cherezov, K. Melcher and H. E. Xu, *Scientific Data*, 2016, **3**, 160021.
